# Molecular Targets in Alveolar Rhabdomyosarcoma: A Narrative Review of Progress and Pitfalls

**DOI:** 10.3390/ijms26115204

**Published:** 2025-05-28

**Authors:** Barbara Ziemba, Klaudia Lukow

**Affiliations:** Department of General Biophysics, Faculty of Biology and Environmental Protection, University of Lodz, 141/143 Pomorska St., 90-236 Lodz, Poland; klaudia.lukow@edu.uni.lodz.pl

**Keywords:** alveolar rhabdomyosarcoma (ARMS), *PAX3-FOXO1*, targeted therapy, receptor tyrosine kinases, transcription factors, therapeutic resistance

## Abstract

Alveolar rhabdomyosarcoma (ARMS) is a highly aggressive pediatric soft-tissue sarcoma driven by *PAX3/7-FOXO1* fusion proteins. Despite intensive multimodal therapy, outcomes remain poor for patients with fusion-positive ARMS. This review integrates recent advances in the molecular pathogenesis of ARMS, highlighting key diagnostic and therapeutic targets. We discuss the central role of fusion proteins in transcriptional reprogramming, impaired myogenic differentiation, and super-enhancer activation. Emerging biomarkers (*YAP*, *TFAP2B*, P-cadherin) and oncogenic kinases (Aurora A, *CDK4*, PLK1) are evaluated alongside receptor tyrosine kinases (*FGFR*, *MET*) and transcription factors involved in metabolic rewiring (*FOXF1*, *ETS1*). Additionally, we examine immunotherapeutic strategies, epigenetic modifiers, and noncoding RNAs as potential therapeutic avenues. Together, these insights provide a comprehensive framework for developing biomarker-guided, multi-targeted therapies to improve outcomes in ARMS.

## 1. Introduction

Rhabdomyosarcoma (RMS) is the most common soft-tissue sarcoma in children and adolescents, accounting for 5–8% of all pediatric malignancies [1,2]. Approximately 70% of RMS cases are diagnosed before the age of 10, although the disease can also occur in adolescents and adults. RMS arises from primitive mesenchymal cells with the potential to differentiate into skeletal muscle and may develop in a wide variety of anatomical locations, including the head and neck region, genitourinary tract, extremities, and trunk [3].

Despite its unified histogenesis, RMS is a clinically and molecularly heterogeneous disease. According to the 2013 World Health Organization (WHO) classification, RMS includes four major histological subtypes: embryonal (ERMS), alveolar (ARMS), pleomorphic (PRMS), and spindle cell/sclerosing RMS (SRMS) [4]. However, recent advances in molecular profiling have refined this categorization, identifying new molecular subtypes such as *MYOD1*-mutated RMS, *VGLL2/NCOA2*-rearranged RMS, and *TFCP2*-rearranged RMS [1,5]. Stratification into these subtypes has significant implications for prognosis and therapeutic decision-making [3,5].

Among these, fusion-positive alveolar RMS is characterized by the presence of *PAX3-FOXO1* or *PAX7-FOXO1* gene fusions, associated with aggressive clinical behavior, early metastasis, and poor outcomes. The five-year overall survival (5y-OS) rate for high-risk FP-RMS remains below 30% [6,7,8]. Histologically, ARMS typically presents an alveolar pattern composed of fibrous septa and discohesive tumor cell nests, with solid and anaplastic variants also reported [4,9,10,11,12].

Multimodal therapy, introduced in the 1970s, combining surgery, radiotherapy, and cytotoxic chemotherapy, has substantially improved survival in patients with localized RMS. VAC (vincristine, actinomycin D, cyclophosphamide) in North America and IVA (ifosfamide, vincristine, actinomycin D) in Europe remain standard chemotherapy regimens [1,8,13]. However, outcomes for patients with metastatic or relapsed ARMS remain unsatisfactory, as current regimens have not significantly improved survival rates in this subgroup [14,15,16].

Encouraged by the success of targeted therapies in other malignancies, recent efforts have focused on identifying molecular vulnerabilities in RMS [17]. Early-phase clinical trials have explored immunotherapeutic strategies and targeted agents in RMS, including anti-*IGF1R* antibodies, receptor tyrosine kinase (RTK) inhibitors, and epigenetic regulators [1,17,18]. While preclinical data have shown promise, clinical translation has remained limited, highlighting the need for a deeper molecular understanding and more refined, biomarker-guided therapeutic approaches.

This review summarizes the current knowledge on molecular targets in FP-RMS, with a focus on transcriptional regulators, kinase signaling networks, epigenetic modulators, and mechanisms of therapeutic resistance.

## 2. *PAX3/7-FOXO1* Fusion Proteins as Master Regulators of ARMS Oncogenesis

ARMS is a pediatric soft-tissue sarcoma characterized by profound genetic alterations that disrupt key cellular processes, including proliferation, apoptosis, differentiation, and epigenetic homeostasis. A hallmark of ARMS is the presence of chromosomal translocations, most commonly t(2;13)(q35;q14) and t(1;13)(q36;q14), which give rise to the chimeric transcription factors *PAX3-FOXO1* and *PAX7–FOXO1*, respectively [12,13]. These fusion proteins are detected in 70–80% of ARMS cases and are strongly associated with a poor prognosis [19,20,21].

Beyond these fusions, other molecular changes have been observed in ARMS. For instance, amplification of the 12q13–q14 chromosomal region containing the *CDK4* proto-oncogene is linked to aggressive subsets of fusion-positive tumors [22], while the tumor suppressor *CAV1* shows little-to-no expression in ARMS [23]. Although rare, alternative *PAX* fusions such as *PAX3–INO80D*, *PAX3–NCOA1*, *PAX3–NCOA2*, and *PAX3–FOXO4* have also been reported in isolated cases [24,25,26].

The *PAX3/7–FOXO1* fusion proteins play a pivotal role in defining the molecular and histologic identity of ARMS. These chimeric proteins act as oncogenic transcription factors with transcriptional potency far exceeding that of their wild-type counterparts, driving target gene expression by 10–100-fold [18]. Notably, *PAX3-FOXO1* overexpression is regulated at the transcriptional level without gene amplification, while *PAX7–FOXO1* overexpression arises from gene amplification [27]. These fusion proteins bind to thousands of genomic loci and activate hundreds of genes involved in tumorigenesis [28]. Key targets include receptor tyrosine kinases (*FGFR2*, *FGFR4*, *IGF1R*, *ALK*, *MET*) and G protein-coupled receptors (*CB1*, *ADRA2C*) [29]. In addition, *PAX3-FOXO1* directly regulates tumor-suppressor genes, such as *GREM1* and *DAPK1*, and indirectly represses *HEY1* through *MYCN* activation [30].

Despite the central role of the fusion proteins in ARMS pathogenesis, they are insufficient alone for full oncogenic transformation [31,32]. Cooperative alterations—such as CDKN2A deletion [33,34,35], TP53 mutation, or promoter methylation [33,36]—are frequently involved. These events contribute to the dysregulation of p16 and p53, leading to altered fusion protein phosphorylation, subcellular localization, and stability [37].

**Figure 1:**
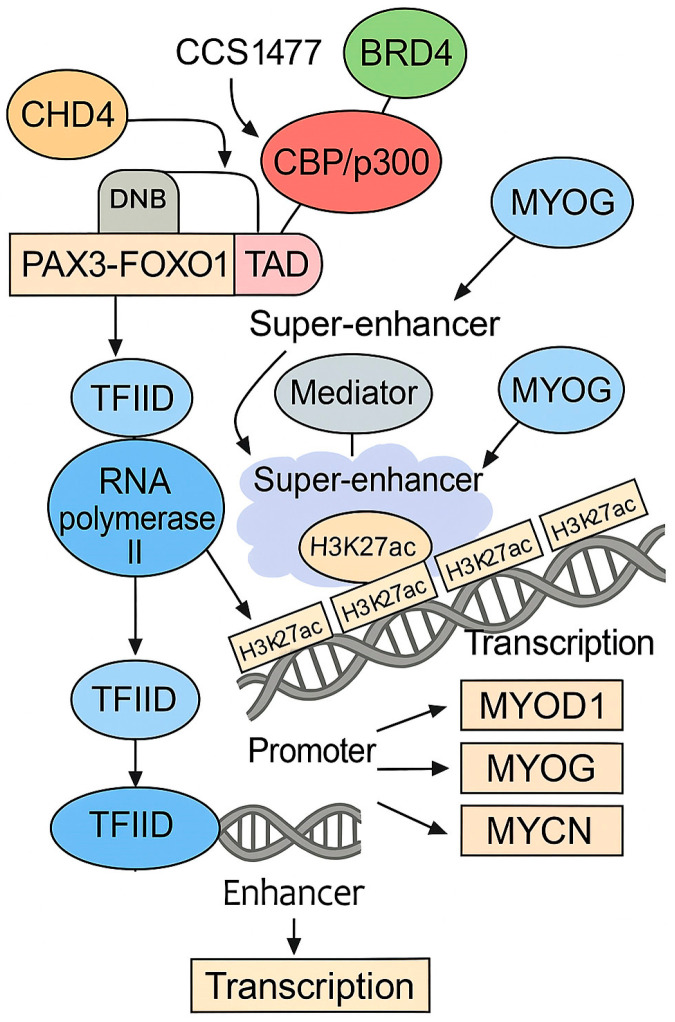
*PAX3-FOXO1*-mediated transcriptional activation in fusion-positive alveolar rhabdomyosarcoma (ARMS). The schematic illustrates how *PAX3-FOXO1* binds enhancer regions and recruits epigenetic regulators such as CHD4, CBP/p300, and BRD4. These interactions drive super-enhancer formation, enhancer–promoter looping via the Mediator complex, and RNA-polymerase-II recruitment. Key target genes, including *MYOD1*, *MYOG*, and *MYCN*, constitute a core transcriptional circuit that sustains the tumorigenic phenotype [39,40]. Key Molecular Players: These comprise *PAX3-FOXO1*, a fusion oncoprotein with DNA-binding (DNB; DNA binding domain) and transactivation (TAD; transcription factor scaffold domain) functions; CBP/p300, histone acetyltransferases recruited via C793 in *FOXO1*; BRD4, which binds acetylated histones and facilitates RNA-Pol-II elongation; CHD4, a chromatin remodeler required for *PAX3-FOXO1* function; Mediator, which promotes enhancer–promoter communication; TFIID and RNA Pol II, components of the transcription initiation complex; and the target genes: *MYOD1*, *MYOG*, *MYCN*, and *FGFR4*.

Importantly, *PAX3-FOXO1* acts as a pioneer factor, reprogramming the chromatin landscape by establishing super-enhancers (SEs) that drive the expression of oncogenic transcriptional networks. This includes auto-regulatory loops involving *MYOG*, *MYOD*, and *MYCN*, which are critical for maintaining the tumorigenic phenotype [37,38,39]. For full function, *PAX3-FOXO1* cooperates with BRD4, a BET bromodomain protein, rendering ARMS cells highly dependent on BRD4 activity [39]. CBP/p300 histone acetyltransferases are key co-activators recruited by the transactivation domain of *PAX3-FOXO1*, promoting local chromatin relaxation via H3K27 acetylation. This epigenetic mark enhances BRD4 binding and supports the assembly of transcriptional condensates at super-enhancer regions. In parallel, the Mediator complex facilitates enhancer–promoter looping and stabilizes the pre-initiation complex, enabling the efficient transcriptional activation of oncogenic targets [39,40]. A schematic representation of this transcriptional activation mechanism is shown in Figure 1.

The fusion protein also influences receptor tyrosine kinase (RTK) expression and perturbs the RTK/RAS/PIK3CA axis. However, similar alterations are present in fusion-negative rhabdomyosarcoma (FN-RMS), suggesting shared downstream mechanisms across RMS subtypes [24]. Regardless of fusion status, frequent activation of PI3K and RAS signaling pathways contributes to dysregulated cell proliferation, survival, and metabolic activity in RMS [14,37,41].

In ARMS, *PAX3-FOXO1*-driven activation of SEs initiates transcriptional programs that sustain malignant cell identity. The resulting core regulatory transcription factors (CR TFs) orchestrate gene expression programs central to tumor growth, positioning ARMS as a compelling model for studying cancer-cell reliance on SE-dependent transcriptional networks [42].

The *PAX3/7–FOXO1* fusion proteins are master regulators of oncogenic transcription in ARMS. By driving super-enhancer formation and modulating the expression of critical transcription factors and signaling components, they reshape the transcriptional and epigenetic landscape of tumor cells. While indispensable for ARMS development, these fusion proteins act in concert with additional genetic alterations to achieve full malignancy. Understanding the molecular circuitry surrounding *PAX3/7–FOXO1* provides a foundation for identifying potential therapeutic vulnerabilities in ARMS. A summary of the key molecular alterations and potential therapeutic targets discussed in this section is presented in Table 1.

## 3. Molecular Diagnostics and Biomarker Identification in Rhabdomyosarcoma

As up to 37.7% of RMS patients may develop distant metastases [43], early and accurate diagnostic tools are crucial for improving clinical outcomes. Exploring the molecular underpinnings of RMS can help identify novel biomarkers for diagnosis and targeted therapy.

Chen et al. [44] performed a transcriptomic analysis comparing RMS and healthy tissues, identifying 164 upregulated and 394 downregulated genes in tumors. Gene-ontology analysis revealed enrichment in pathways related to the cell cycle, muscle contraction, cytoskeletal organization, and nucleotide binding—reflecting disrupted proliferation and myogenic differentiation in RMS.

A protein–protein interaction (PPI) network comprising 441 nodes and 3274 edges led to the identification of ten hub genes. Among them, *MYBPC2* and *MYL1* were significantly upregulated in RMS and associated with worse overall survival, highlighting their potential prognostic value.

These results suggest that *MYBPC2* and *MYL1* contribute to RMS pathogenesis and may serve as promising biomarkers for early diagnosis and risk stratification, warranting further investigation in clinical settings.

### 3.1. Diagnostic Implications of YAP Activation in Alveolar Rhabdomyosarcoma

*PAX3-FOXO1* drives key genetic and epigenetic changes, including the evasion of cellular senescence in human skeletal muscle myoblasts (HSMMs), partly through the upregulation of *RASSF4* [35]. *RASSF4* inhibits *MST1*, a core Hippo pathway kinase, leading to enhanced cell-cycle progression and tumorigenesis [45].

Suppression of the Hippo pathway results in the activation of Yes-associated protein 1 (*YAP*), a transcriptional co-regulator that promotes proliferation. Although *YAP* activity is increased in RMS, immunohistochemistry reveals a characteristic pattern in ARMS: nuclear *YAP* staining is typically limited to <30% of tumor cells [46]. This reflects the dynamic regulation of *YAP*, whose localization fluctuates in response to signals such as mechanical stress, cell density, and polarity. These factors influence *YAP*’s shuttling between cytoplasm and nucleus, resulting in heterogeneous nuclear expression [47].

This focal nuclear staining pattern distinguishes ARMS from other RMS subtypes, such as embryonal and spindle-cell RMS, which show diffuse nuclear YAP positivity. Therefore, limited nuclear *YAP* expression may serve as a supportive diagnostic marker for ARMS and underscores the broader relevance of Hippo-*YAP* pathway dysregulation in its pathogenesis.

### 3.2. The Central Role of PAX3-FOXO1 in ARMS Pathogenesis and Diagnostics

Accurate subclassification of rhabdomyosarcoma is clinically essential, especially given the poor prognosis and aggressive therapeutic regimens required for patients with FP-RMS. The detection of the *PAX3-FOXO1* or *PAX7–FOXO1* gene fusion is, therefore, critical for both diagnostic and therapeutic decisions. Traditionally, molecular methods such as fluorescence in situ hybridization, RT-PCR, or next-generation sequencing are employed, but these approaches are often complex and time-consuming. To overcome this, Azorsa et al. [48] developed monoclonal antibodies (PFM.1 and PFM.2) specific to the junction region of the *PAX3-FOXO1* fusion protein. These antibodies showed high specificity and sensitivity in immunohistochemical analyses of tumor sections, enabling rapid and reliable detection of fusion proteins in tissue samples [48]. This advance offers a valuable tool for routine diagnostics, complementing molecular assays and underscoring the clinical importance of identifying FP-RMS.

Functionally, the *PAX3/7–FOXO1* fusion proteins are central oncogenic drivers in FP-RMS. Genetic loss-of-function studies have demonstrated that tumor-cell survival is strictly dependent on the continued activity of these fusion proteins, which control gene-expression programs essential for maintaining malignancy [21,33,34,35]. Kikuchi et al. [49] used RNA interference to knock down *PAX3-FOXO1* in human ARMS cell lines, leading to decreased proliferation and G1 cell-cycle arrest. This intervention also reduced *MET*-receptor expression, impairing cell motility, and it triggered myogenic differentiation by upregulating genes such as *MYOG* and promoting the expression of muscle-specific proteins like desmin and myosin heavy chain [49]. These results highlight that the direct targeting of *PAX3-FOXO1* not only inhibits tumor growth but may also restore features of normal muscle differentiation, positioning it as a highly attractive therapeutic strategy.

Building upon these findings, Nakazawa et al. [50] employed surface plasmon resonance technology to screen small-molecule libraries for compounds capable of directly binding to the *PAX3-FOXO1* protein. Among the identified hits, piperacetazine—a phenothiazine-derived antipsychotic compound—emerged as a direct binder of the fusion protein. Piperacetazine demonstrated the ability to inhibit *PAX3-FOXO1*-driven transcriptional activity across multiple FP-RMS cell lines. Notably, it selectively impaired anchorage-independent growth in FP-RMS cells, while showing no significant effect in ERMS models.

These findings suggest that piperacetazine is a promising scaffold for developing more potent and selective *PAX3-FOXO1* inhibitors. Its selective efficacy in fusion-positive cells highlights its therapeutic potential. Future work may focus on optimizing its structure to improve specificity and pharmacokinetics, enabling progression to preclinical and clinical testing for recurrent or metastatic FP-RMS [50].

However, monotherapies targeting *PAX3-FOXO1* are not without limitations. Boudjadi et al. [51] observed that, although suppression of the fusion protein initially led to tumor regression, resistant recurrent tumors eventually emerged. These recurrences were driven by the upregulation of fibroblast growth factor 8 (*FGF8*), a direct transcriptional target of *PAX3-FOXO1*. *FGF8* compensated for the loss of the fusion protein, reactivating oncogenic pathways and promoting tumor regrowth. This highlights a key challenge: targeting *PAX3-FOXO1* may be insufficient unless combined with therapies that disrupt downstream effectors such as *FGF8* [51].

Together, these findings emphasize not only the diagnostic value of detecting *PAX3-FOXO1* in FP-RMS but also its pivotal role in tumor maintenance and resistance. Comprehensive therapeutic strategies must, therefore, consider both direct inhibition of the fusion protein and blockade of its downstream oncogenic networks.

### 3.3. Myogenin in Rhabdomyosarcoma: A Diagnostic Marker and a Blocked Driver of Differentiation

Myogenin, a member of the myogenic regulatory factor (MRF) family, plays a pivotal role in the commitment and differentiation of mesenchymal progenitors into the skeletal muscle lineage [52].

Its expression is tightly linked to rhabdomyoblastic differentiation, making it a highly specific immunohistochemical marker in the diagnosis of RMS, particularly for distinguishing RMS from other pediatric small round cell tumors.

In an immunohistochemical analysis using the myf-4 antibody on FFPE tissue, myogenin expression was detected in all tested RMS cases. ARMS showed strong, diffuse nuclear staining in 75–100% of cells, whereas ERMS displayed weaker and more variable expression, usually limited to ≤25% of cells.

These findings highlight the diagnostic value of myogenin as a specific marker of rhabdomyoblastic differentiation. Its widespread expression in ARMS suggests a higher degree of myogenic commitment, whereas the focal pattern in ERMS may indicate variable stages of differentiation. The lack of staining in non-RMS tumors underscores its specificity in pediatric soft-tissue tumor diagnostics [53].

Paradoxically, despite high *MYOG* expression, ARMS cells fail to complete terminal differentiation. This is partly due to the oncogenic *PAX3-FOXO1* fusion protein, which enhances GSK3β activity, leading to phosphorylation and the suppression of MYOGENIN function. In vitro and in RH30 ARMS cells, *MYOG* was shown to be a GSK3β substrate. Mutation of the phosphorylation site (S160/164A) restored *MYOG* activity and reduced tumorigenic growth, highlighting a key mechanism by which differentiation is blocked in ARMS [54].

### 3.4. TFAP2B as a Downstream Effector of PAX3-FOXO1 and Diagnostic Marker in FP-RMS

*TFAP2B* encodes AP-2β, a member of the AP-2 family of transcription factors, which regulate gene expression by forming homo- or heterodimers with other AP-2 family members and binding to specific DNA sequences. These transcription factors are known to modulate key developmental processes by promoting cell proliferation and inhibiting terminal differentiation during embryogenesis [55]. In the context of ARMS, *TFAP2B* operates as a transcriptional target of the oncogenic *PAX3-FOXO1* fusion protein, placing it downstream within the aberrant gene regulatory hierarchy that drives tumorigenesis.

Functionally, *TFAP2B* contributes to the malignant phenotype by transmitting anti-apoptotic signals and promoting cell survival. Gene-silencing experiments using small interfering RNA (siRNA) targeting *TFAP2B* have demonstrated that downregulation of this factor results in a significant reduction in mRNA and protein levels, leading to decreased tumor-cell proliferation and increased apoptosis. These effects mirror those observed upon thevsilencing of *PAX3-FOXO1* itself, reinforcing the role of *TFAP2B* as a critical downstream effector of this fusion oncoprotein [56].

*TFAP2B* has emerged as a valuable ancillary biomarker for the histopathological diagnosis of ARMS, particularly the FP subtype. In biopsy specimens, *TFAP2B* expression serves as a sensitive and specific marker for FP-RMS, especially when used in conjunction with P-cadherin detection [56,57,58]. The diagnostic utility of this combination is notably high, supporting more accurate tumor classification in challenging clinical cases.

Taken together, *TFAP2B* not only represents a robust biomarker for the diagnosis of FP-RMS but also contributes functionally to the disease’s pathogenesis. Its regulatory dependence on *PAX3-FOXO1* and its pro-survival properties position *TFAP2B* as both a diagnostic and potential therapeutic target in ARMS.

### 3.5. P-Cadherin as a Mediator of ARMS Aggressiveness

P-cadherin is a classical cell adhesion molecule essential for tissue homeostasis, yet its role in tumorigenesis is context-dependent. While acting as a tumor suppressor in melanoma and oral squamous cell carcinoma, its overexpression is associated with tumor-promoting functions in several cancers, including breast, ovarian, prostate, endometrial, skin, gastric, pancreatic, and colon malignancies [59].

In ARMS, P-cadherin expression has been linked to the presence of the *PAX3/7-FOXO1A* fusion protein. Studies using dominant-negative PAX3 constructs demonstrated that the inhibition of *PAX3* activity leads to reduced P-cadherin levels, indicating transcriptional regulation. In vivo experiments in *Pax3*-modified mouse models further confirmed P-cadherin expression in the dermomyotome, supporting its position downstream of *Pax3* in the myogenic hierarchy.

Mechanistically, gel shift assays and chromatin immunoprecipitation revealed direct binding of *PAX3/7-FOXO1A* to P-cadherin regulatory elements, establishing it as a direct transcriptional target. Functional studies show that P-cadherin overexpression in normal myoblasts disrupts differentiation and induces transformation, migration, and invasion—hallmarks of malignancy. In contrast, P-cadherin silencing via RNA interference in ARMS cells reverses these aggressive phenotypes.

Additionally, P-cadherin facilitates cadherin switching—a hallmark of metastasis—by modulating the expression and localization of N- and M-cadherins. These findings identify P-cadherin as a downstream effector of *PAX3-FOXO1A*-driven oncogenic signaling in ARMS and suggest it as a potential therapeutic target [60].

Advances in molecular diagnostics have significantly enhanced our understanding of rhabdomyosarcoma, particularly fusion-positive alveolar RMS. Key molecular alterations—such as the *PAX3-FOXO1* fusion, dysregulation of the Hippo-*YAP* pathway, and aberrant expression of biomarkers like *MYBPC2*, *MYL1*, *TFAP2B*, and P-cadherin—offer valuable tools for diagnosis, prognosis, and therapeutic targeting. While novel strategies, including fusion protein-specific antibodies and small-molecule inhibitors, show promise, overcoming resistance mechanisms will require combination approaches. Continued integration of molecular profiling into clinical practice is essential for improving diagnostic precision and patient outcomes.

## 4. Transcriptional Repression of Differentiation Pathways in Fusion-Positive Rhabdomyosarcoma

FP-RMS is characterized by impaired skeletal muscle differentiation and sustained proliferation, driven by specific transcriptional deregulation. This section focuses on three distinct, but interconnected, mechanisms of transcriptional repression that contribute to ARMS pathogenesis: (1) *PAX3-FOXO1*–mediated silencing of *ACTA1* via the RhoA–MKL1–SRF pathway, (2) *TBX2*-driven repression of muscle-specific gene expression, and (3) *MYOG* inactivation and its exploitation in targeted gene therapy. Each of these pathways underscores a critical molecular vulnerability that could be leveraged therapeutically to promote differentiation and suppress tumor growth in ARMS.

### 4.1. PAX3-FOXO1-Mediated Repression of ACTA1 via the RhoA–MKL1–SRF Pathway

The *ACTA1* gene encodes skeletal muscle alpha-actin, a core component of the sarcomeric thin filament in adult skeletal muscle, and one of six highly conserved actin isoforms implicated in human disease [61].

Hu et al. [62] demonstrated that the oncogenic fusion protein *PAX3-FOXO1* suppresses *ACTA1* expression at both the transcriptional and protein levels. This suppression occurs via interference with the RhoA–MKL1–SRF signaling axis. RhoA, a small GTPase, modulates actin dynamics and gene transcription [63]. MKL1 (megakaryoblastic leukemia 1) acts as a coactivator that translocates to the nucleus in response to actin polymerization and interacts with SRF (serum response factor), a transcription factor [64], to activate genes such as *ACTA1*. The small molecule STARS (striated muscle activator of Rho signaling) [65] enhances this pathway, whereas Cytochalasin D, a fungal metabolite [66], disrupts actin filaments and affects signaling indirectly.

Reporter assays showed that *PAX3-FOXO1* overexpression significantly reduced *ACTA1* promoter activation induced by RhoA, MKL1, SRF, STARS, and Cytochalasin D. Furthermore, co-treatment with CCG-1423 [67], an inhibitor of the RhoA–MKL1–SRF pathway, led to a synergistic reduction in *ACTA1* activity. The knockdown of *PAX3-FOXO1* restored *ACTA1* expression, and *PAX3-FOXO1* also reduced MKL1 and SRF protein levels and their interaction, though without altering their subcellular localization [62].

Functionally, the overexpression of *ACTA1* in RH30 ARMS cells reduced proliferation and migration in vitro and impaired tumor growth in vivo, supporting a tumor-suppressive role for *ACTA1* in this context. These findings suggest that the repression of *ACTA1* by *PAX3-FOXO1* through the RhoA–MKL1–SRF axis contributes to ARMS tumorigenesis and progression. As such, restoring *ACTA1* expression or targeting components of this pathway may offer a novel therapeutic approach in the management of ARMS [62].

### 4.2. TBX2 as a Transcriptional Repressor in Rhabdomyosarcoma

The T-box transcription factor TBX2 is markedly upregulated in tumor cells across both major RMS subtypes—ARMS and ERMS. Known for its role as a transcriptional repressor, TBX2 contributes to oncogenesis by bypassing normal cell-cycle checkpoints, in part through the suppression of *CDKN2A (*p14^ARF^*)* and *CDKN1A (*p21^CIP1^*)*, key regulators of cell-cycle arrest and differentiation [68]. Notably, p21, essential for terminal skeletal muscle differentiation [69], is silenced in RMS cells.

Mechanistically, TBX2 interacts with myogenic regulatory factors such as MyoD and myogenin, inhibiting their transcriptional activity. It also recruits histone deacetylase 1 (HDAC1), reinforcing the repression of muscle-specific genes and promoting a proliferative state. Importantly, TBX2 expression decreases significantly upon myogenic differentiation.

Functional studies have shown that depletion or dominant-negative inhibition of TBX2 leads to the reactivation of *p21* and muscle-specific genes, impairing tumor growth. In xenograft models, TBX2 inhibition completely blocked tumor development, underscoring its oncogenic potential in RMS [70].

Together, these findings position TBX2 as a critical factor sustaining the undifferentiated, proliferative phenotype of RMS cells. Its role as a transcriptional repressor of differentiation-specific programs makes it a compelling candidate for diagnostic and therapeutic targeting in RMS.

### 4.3. Myogenin-Driven Targeted Gene Therapy in ARMS

Building upon the molecular specificity of myogenin, therapeutic strategies have explored the use of myogenin regulatory elements in targeted gene therapy for ARMS. Pruller et al. [13] engineered a minimal *MYOG* promoter [52] by deleting NF1 and MEF3 transcription-factor binding sites, creating an ARMS-specific promoter that robustly drives expression in ARMS cells but not in normal skeletal myoblasts. This promoter was used to control the expression of the herpes simplex virus thymidine kinase (HSV-TK) suicide gene, resulting in efficient ARMS cell killing in vitro. Furthermore, in a xenograft mouse model, the ARMS promoter-HSV-TK construct induced the apoptosis of ARMS cells in vivo. Combining this therapy with conventional chemotherapeutics allowed for dose reduction, potentially minimizing toxicity and treatment burden. This ARMS-specific promoter offers a promising approach to targeted gene therapy in FP-RMS, aiming to improve outcomes in this aggressive cancer.

The disruption of myogenic differentiation through transcriptional repression is a hallmark of rhabdomyosarcoma biology. Both *PAX3-FOXO1* and TBX2 act to suppress key regulators of the muscle program—*ACTA1* and *CDKN1A* (*p21*)—via distinct yet complementary mechanisms. These findings emphasize the importance of reactivating differentiation pathways as a therapeutic strategy in ARMS and supporting the further development of targeted gene therapies and pathway-specific inhibitors to reverse oncogenic transcriptional repression.

## 5. The Role of Serine/Threonine-Protein Kinases in the Development and Treatment of ARMS

Serine/threonine-specific protein kinases are enzymes that phosphorylate the hydroxyl groups of serine or threonine residues in target proteins, thereby modulating their activity, localization, stability, or interactions. As key components of intracellular signaling pathways, including MAPK/ERK and PI3K/AKT, they regulate essential cellular processes such as proliferation, differentiation, metabolism, and apoptosis. Aberrant kinase activity is frequently implicated in the development of cancer and other diseases, making these kinases critical therapeutic targets [71].

### 5.1. Targeting Aurora A (AurA) to Disrupt PAX3-FOXO1 Function and Enhance FP-RMS Therapy

Ommer et al. [72] proposed a combination therapy targeting *PAX3-FOXO1* on multiple levels. Their research showed that the pharmacological depletion of *PAX3-FOXO1* in FP-RMS cells triggered intrinsic apoptosis via NOXA activation. In parallel, the inhibition of Aurora A kinase—essential for mitotic progression [73]—led to a marked reduction in *PAX3-FOXO1* protein levels, due to a newly identified phosphorylation-dependent binding mechanism. Aurora A was also shown to stabilize MYCN, a key oncogenic driver and transcriptional target of *PAX3-FOXO1*. While all Aurora kinases promote mitosis, Aurora A uniquely supports tumor stemness, epithelial–mesenchymal transition, and invasion. The co-inhibition of *PAX3-FOXO1* and Aurora A synergistically triggered apoptosis and significantly delayed tumor growth in FP-RMS cell lines and xenograft models [72].

Building on these findings, Ommer et al. further demonstrated that Aurora A directly binds and phosphorylates *PAX3-FOXO1* at Ser^437^—a modification that protects the fusion protein from proteasomal degradation and sustains its oncogenic activity. Pharmacologic inhibition of Aurora A disrupts this protective mechanism, resulting in *PAX3-FOXO1* destabilization and apoptotic cell death. Thus, Aurora A functions as a post-translational stabilizer of *PAX3-FOXO1* and represents a critical therapeutic node for fusion-protein-directed intervention [72].

### 5.2. CDK4 Amplification and—Targeting in FP-RMS

The amplification of the chromosomal region 12q13–q14, which harbors the *CDK4* proto-oncogene, has been identified as a recurrent alteration in an aggressive subset of fusion-positive rhabdomyosarcoma (RMS) [74,75]. The oncogenic potential of this amplification stems from dysregulated cell-cycle progression via the CDK4–RB–E2F axis, a critical regulatory pathway in many cancers.

Preclinical studies have demonstrated the antiproliferative efficacy of CDK4/6 inhibitors in *CDK4*-amplified malignancies, including well-differentiated and dedifferentiated liposarcoma [76] and neuroblastoma [77]. These findings have prompted investigation into the role of CDK4/6 inhibition as a therapeutic strategy in FP-RMS.

Functional studies revealed that the knockdown of CDK4 in both 12q13–14-amplified and non-amplified FP-RMS cell lines leads to a marked reduction in proliferation and transformation. This effect was mediated via G1-phase cell-cycle arrest, associated with decreased RB phosphorylation and the downregulation of E2F-responsive gene expression. Notably, the overexpression of CDK4 alone did not replicate these effects, indicating that, while CDK4 is necessary for RB–E2F-mediated G1 progression and cellular proliferation, its overexpression is not sufficient to drive transformation in FP-RMS [22].

Pharmacologic inhibition using the selective CDK4/6 inhibitor LEE011 (ribociclib [78]) phenocopied the genetic knockdown effects, inducing G1 arrest, reducing RB phosphorylation, and diminishing E2F target-gene expression. Importantly, although all of the tested FP-RMS cell lines responded to ribociclib treatment, the degree of sensitivity inversely correlated with CDK4 expression levels. Cells harboring *CDK4* amplification exhibited relatively reduced sensitivity, suggesting a complex relationship between gene dosage and drug responsiveness. This variable sensitivity was also observed in in vivo xenograft models, further supporting the differential antitumor activity based on *CDK4* status [22].

In addition to its role in driving RB–E2F-mediated cell-cycle progression, CDK4 has also been shown to modulate *PAX3-FOXO1* function directly. Specifically, CDK4 phosphorylates *PAX3-FOXO1* at Ser^430^, a modification that enhances the transcriptional activity of the fusion protein [79]. This finding suggests that CDK4/6 inhibitors may disrupt not only proliferation but also the oncogenic transcriptional program driven by *PAX3-FOXO1*. Accordingly, the pharmacological blockade of CDK4/6 may exert dual antitumor effects in FP-RMS by inducing G1 arrest and dampening fusion-protein-dependent gene expression.

Collectively, these data support a model in which CDK4 activity is essential for RMS cell proliferation but highlight that high CDK4 expression may attenuate sensitivity to CDK4/6 inhibition. This paradoxical observation underscores the need for biomarkers beyond mere *CDK4* amplification status to predict therapeutic response.

The preclinical evidence has catalyzed early-phase clinical evaluations of ribociclib in pediatric solid tumors. A Phase I trial investigated the maximum tolerated dose (MTD), recommended Phase II dose (RP2D), safety, pharmacokinetics (PK), and the preliminary efficacy of single-agent ribociclib in children with cyclin D–CDK4/6–INK4–RB pathway-altered tumors, including RMS and neuroblastoma. The study reported acceptable safety and PK profiles, with pediatric MTD (470 mg/m^2^) and RP2D (350 mg/m^2^) aligning with adult dosing parameters. Although objective responses were limited, prolonged stable disease (SD) in several patients supports the rationale for combining ribociclib with cytotoxic or targeted agents to enhance efficacy [80].

Building on this foundation, a Phase I/II clinical trial (NCT05429502) [81] is currently underway to evaluate the combination of ribociclib with topotecan and temozolomide (TOTEM regimen) in pediatric patients with relapsed or refractory neuroblastoma and other solid tumors, including medulloblastoma, high-grade glioma (HGG), malignant rhabdoid tumors (MRT), and RMS. This study aims to assess the safety, tolerability, and preliminary efficacy of ribociclib in a combinatorial setting, potentially expanding therapeutic options for these challenging malignancies.

Together, these findings provide compelling preclinical and early clinical evidence supporting the continued investigation of CDK4/6-targeted therapies in FP-RMS. Further studies are warranted to define the optimal therapeutic context, identify predictive biomarkers of response, and determine the role of combination strategies in enhancing treatment outcomes.

### 5.3. Polo-like Kinase 1 (PLK1) as a Therapeutic Target in ARMS

Polo-like kinase 1 (PLK1) is a serine/threonine-specific kinase that plays a pivotal role in the regulation of mitotic progression and the cellular response to DNA damage. In RMS, particularly the alveolar subtype, PLK1 is frequently overexpressed and its high expression levels are associated with poor prognosis, highlighting its potential as a molecular target for therapeutic intervention [82].

Initial evidence supporting PLK1 as a therapeutic target in ARMS emerged from genome-wide siRNA library screens conducted by Hu et al. [83], who identified PLK1 as the most potent regulator of proliferation in the ARMS cell line RH30. Silencing PLK1 led to approximately 80% growth inhibition and the induction of apoptosis in RMS cells, with no adverse effects observed in normal muscle cells. Moreover, PLK1 mRNA was found to be overexpressed in 47% of the primary ARMS tumors analyzed [83]. At the molecular level, PLK1 knockdown disrupted the G2/M transition by downregulating phosphorylation of CDC25C and Cyclin B1, while upregulating WEE1, a known mitotic checkpoint regulator [84,85].

The oncogenic *PAX3-FOXO1* fusion protein, a defining molecular hallmark of ARMS, has also been linked to PLK1 signaling. Thalhammer et al. [86] demonstrated that PLK1 phosphorylates *PAX3-FOXO1* at a previously unidentified S503 site, stabilizing the protein and sustaining its oncogenic activity. The pharmacological inhibition of PLK1 using BI-2536-enhanced ubiquitination and proteasomal degradation of *PAX3-FOXO1*, reduced its target gene expression, and induced tumor regression in preclinical models [86]. These findings provide strong evidence for a functional PLK1–PAX3-FOXO1 axis in ARMS pathogenesis and validate PLK1 as a rational therapeutic target.

Despite these promising results, monotherapy with PLK1 inhibitors has shown limitations due to the development of tumor regrowth, underscoring the need for rational combination strategies. Gatz et al. [82] reported that low-dose volasertib (BI 6727), a potent PLK1 inhibitor, exhibits synergistic antitumor effects when combined with vincristine, a microtubule-targeting agent widely used in RMS treatment. This combination improved treatment outcomes in preclinical models and, importantly, is supported by pharmacokinetic data indicating achievable therapeutic levels in pediatric patients.

Further evidence for PLK1-targeted combination therapies comes from studies involving Eribulin, another microtubule-disrupting agent with demonstrated efficacy in pediatric solid tumors. Stehle et al. [87] showed that co-treatment with Eribulin and the PLK1 inhibitor BI 2536 at subtoxic concentrations synergistically induced apoptosis in RMS cell lines and patient-derived ARMS cultures, significantly reducing cell viability and colony formation. The combination also suppressed tumor growth in vivo. Mechanistically, the antitumor synergy depended on sustained mitotic arrest, as pharmacological inhibition of CDK1 abrogated the effect. Apoptosis was mediated via both caspase-dependent and caspase-independent pathways, including the activation of endonuclease G. Importantly, similar effects were observed in neuroblastoma models and with other drug combinations such as vincristine and Poloxin, suggesting a broader relevance of dual mitotic targeting strategies in pediatric oncology.

Taken together, preclinical evidence strongly supports PLK1 as a valid and promising molecular target in ARMS. Its role in stabilizing the *PAX3-FOXO1* oncoprotein and regulating mitotic progression makes it uniquely positioned for therapeutic exploitation. Combination regimens involving PLK1 inhibitors and microtubule-targeting agents such as vincristine or Eribulin appear particularly promising and warrant further investigation in clinical settings.

### 5.4. ERK Signaling as a Therapeutic Target in RMS

The RAS–RAF–MEK–ERK signaling cascade is a key regulator of proliferation, differentiation, migration, and survival in both normal and cancerous cells. Aberrant activation of this pathway, particularly through sustained ERK1/2 activity, contributes to malignant transformation by promoting uncontrolled proliferation, immortalization, and impaired differentiation. ERK1 and ERK2 are serine/threonine kinases activated by dual phosphorylation at Thr202/185 and Tyr204/187 by MEK1/2, a modification essential for their full activity [88]. Although structurally related, ERK1 and ERK2 can have distinct functional roles depending on the cellular context.

Otabe et al. [89] demonstrated that in ARMS, HGF/MET signaling promotes cell motility predominantly via ERK2. The selective inhibition of ERK2 phosphorylation reduced invasiveness and metastasis, while ERK1 inhibition or knockdown had minimal impact, suggesting ERK2 as a more promising therapeutic target in ARMS. Moreover, dual targeting of ERK1 and mTOR did not significantly reduce HGF-induced motility, further supporting the specificity of ERK2 involvement.

In a related study, the role of ERK activity in regulating differentiation was examined in SH-SY5Y neuroblastoma, U-251 astrocytoma, and TE-671 rhabdomyosarcoma cells using a live-cell reporter system to monitor *ERK* dynamics [90]. Selective ERK inhibition, unlike retinoic acid, led to marked morphological changes in TE-671 cells and the upregulation of myogenic differentiation markers *PROM1*, *MYOG*, and *PAX7*. These changes were closely linked to ERK activity at the single-cell level, while retinoic acid-induced differentiation in SH-SY5Y cells occurred independently of ERK. In addition, ERK inhibition sensitized TE-671 cells to EGF, IGF-1, and NGF, likely through the suppression of basal ERK activity and the enhanced response to *BDNF* via increased *TrkB*-receptor expression.

Aberrant ERK signaling is also a therapeutic vulnerability in FN-RMS, where *RAS* mutations (e.g., *H/NRAS^Q61X^*) drive tumor growth. Garcia et al. [91] confirmed the central role of the ERK MAPK pathway in mediating *RAS* dependency in FN-RMS. However, treatment with the MEK inhibitor trametinib achieved only modest in vivo efficacy due to the incomplete suppression of ERK activity. CRISPR screening identified CRAF as a key mediator of *ERK*-pathway reactivation following MEK or ERK inhibition. To overcome this limitation, vertical inhibition strategies co-targeting MEK with either ERK or CRAF—or using pan-RAF inhibitors (pan-RAFi) in combination with MEK or ERK inhibitors (MEKi or ERKi)—were evaluated. Combinations such as MEKi + ERKi and pan-RAFi + ERKi synergistically blocked ERK activity, induced differentiation and apoptosis, and significantly reduced tumor growth in xenograft models, with pan-RAFi + ERKi showing the greatest efficacy and tolerability.

Despite the promise of ERK-pathway inhibition, its blockade alone is often insufficient to induce robust cell death in RMS due to compensatory survival signals, particularly from the anti-apoptotic protein MCL-1. The co-inhibition of ERK1/2 (using Ulixertinib) and MCL-1 (using S63845) triggered mitochondrial apoptosis via rapid caspase activation and effectively suppressed RMS cell survival both in vitro and in vivo [92]. Notably, this effect was independent of fusion status, highlighting the broad therapeutic potential of this combination strategy.

Collectively, these findings underscore the central role of ERK signaling in RMS pathogenesis and point to the therapeutic benefit of dual or vertical inhibition strategies, particularly those targeting ERK2, CRAF, or MCL-1. Such approaches may overcome resistance mechanisms and enhance differentiation and apoptosis in both FP- and FN-RMS.

### 5.5. ATR Inhibition in ARMS: Exploiting DNA Damage Response Vulnerabilities

Targeting the DNA damage response (DDR) represents a promising therapeutic approach in cancer, offering potential for tumor-selective treatment. Among the central regulators of the DDR are ataxia telangiectasia and Rad3-related protein (ATR) and its key downstream effector, checkpoint kinase 1 (CHK1). Cancer cells are particularly reliant on the ATR-CHK1 pathway, providing a strong rationale for the therapeutic targeting of these kinases [93].

FP-RMS cells display heightened sensitivity to the pharmacological inhibition of ATR [94], a serine/threonine-protein kinase essential for preserving genome stability through the DNA damage response [95]. The expression of the *PAX3-FOXO1* fusion oncogene further increases susceptibility to ATR inhibition, and FP-RMS cells respond consistently to structurally diverse ATR inhibitors, underscoring a robust therapeutic dependency.

Mechanistically, an ATR blockade exacerbates replication stress, reduces BRCA1 phosphorylation, and impairs homologous recombination-mediated DNA repair. This disruption sensitizes ARMS cells to *PARP1* inhibition, with the combined targeting of ATR and PARP1 resulting in synergistic cytotoxicity in vitro and the complete regression of patient-derived xenografts in vivo. Resistance to *ATR* inhibition has been associated with the activation of the RAS-MAPK signaling cascade and the *FOS* gene family [94]. Collectively, these findings establish ATR as a critical therapeutic target in ARMS and support the rationale for biomarker-driven clinical trials.

Although the ATR-CHK1 axis has long been the subject of intensive research, the clinical development of ATR inhibitors has only recently progressed, e.g., NCT04095221 [96] and more [97,98,99,100].

### 5.6. Targeting PAK4 in RMS: Inhibition of Oncogenic Signaling and Metastasis

Dasgupta et al. [101] indicate p21-activated kinase 4 (PAK 4) as a viable therapeutic target for advanced RMS treatment. They demonstrated that knocking down or inhibiting PAK4 reduces cell proliferation in vitro and delays or regresses tumor growth in vivo. What is more, blocking PAK4 had a profound effect on metastasis [101].

PAK4 is a member of the PAK family, which regulates a wide range of cellular functions, including cell adhesion, migration, proliferation, and survival. Dysregulation of its expression and activity contributes to the development of diverse pathological conditions [102]. PAKs are downstream of multiple critical tumorigenic RTK and oncogenic regulators, including IGFR and RAS signaling [101].

PAK4 signaling supports the growth of cancer cells through AKT- and ERK-regulated signaling. Its upregulation in cancer cells leads to increased cell survival, anchorage-independent growth, and transformation in experimental models. The overexpression of PAK4 correlates with poor prognosis, tumor aggressiveness, metastasis, and infiltration [103]. It is regarded as an attractive therapeutic target in diverse cancer types, prompting the development of PAK4-specific inhibitors as anticancer drugs [102].

PAK4 exhibits enhanced expression levels and activity in both ARMS and ERMS. In Dasgupta et al.’s [101] research, several primary and metastatic in vivo models, including a relapsed RMS patient-derived xenograft model, were sensitive to PAK4 small-molecule inhibitors, i.e., PF-3758309 and KPT-9274. The molecular perturbation of PAK4 resulted in RAS-GTPase, Hedgehog, and Notch pathways inhibition, along with the activation of an antitumor immune response [101].

Serine/threonine-protein kinases play a pivotal role in the oncogenic landscape of alveolar rhabdomyosarcoma (ARMS), governing key processes such as cell-cycle progression, mitosis, DNA damage repair, and survival signaling. The aberrant activity of kinases such as Aurora A, CDK4, PLK1, ERK1/2, ATR, and PAK4 contributes to tumorigenesis, therapeutic resistance, and metastasis. Preclinical and early clinical studies have demonstrated that selective inhibition of these kinases—either as monotherapy or in rational combinations—can suppress tumor growth, induce apoptosis, promote differentiation, and sensitize ARMS cells to conventional therapies.

Importantly, the therapeutic efficacy of kinase inhibition is context-dependent and often influenced by fusion status, gene dosage, pathway redundancy, and compensatory signaling. As such, future research should prioritize the identification of predictive biomarkers, optimization of combination regimens, and development of next-generation inhibitors with enhanced selectivity and tolerability. Together, these efforts may lead to more effective and durable treatment strategies for patients with ARMS.

## 6. Transcription Factors in Metabolic Reprogramming and Cancer

Metabolic reprogramming is a hallmark of cancer, supporting proliferation, survival, and therapy resistance by altering energy production and signaling metabolite synthesis. Transcription factors (TFs) play a central role in regulating these processes and are frequently deregulated in cancer, impacting both metabolism and gene expression [104]. Despite advances, understanding how TFs specify genomic binding and influence transcription remains a major challenge [105]. TFs also govern key developmental pathways, and their dysregulation can drive malignant transformation. Conserved TF families such as HMG, GATA, PAX, and bHLH are implicated in both embryogenesis and tumorigenesis, underscoring their relevance as potential therapeutic targets [106].

### 6.1. FOXF1 as a Therapeutic Target in Rhabdomyosarcoma

The *FOXF1* (*Forkhead box F1*) gene, located on chromosome 16q24.1, encodes a transcription factor of the FOX family, defined by a conserved forkhead DNA-binding domain. Acting downstream of the Sonic Hedgehog (Shh) pathway, FOXF1 is essential for epithelium–mesenchyme interactions during development [107]. Mounting evidence highlights *FOXF1*’s oncogenic role in rhabdomyosarcoma (RMS), particularly in the alveolar subtype (ARMS), making it a promising therapeutic target.

Gene-expression profiling in RMS tissues identified 211 upregulated genes in ARMS, including *FOXF1* and *LMO4*, especially in metastatic cases. These transcription factors regulate cell growth and motility, and their silencing reduced RMS cell invasion more than ten-fold, indicating their contribution to metastatic behavior [108].

Mechanistic studies have demonstrated that FOXF1 and its paralog FOXF2 are crucial for RMS tumor growth. Their knockdown in mouse models significantly inhibited tumor development due to decreased cell proliferation. The loss of FOXF1/FOXF2 altered the expression of key cell-cycle regulators (*CDK2*, *CDK4*, *CDK6*, *CCND1*, *CCNE2*), reduced Rb phosphorylation, and increased p21^Cip1^ and p27^Kip1^ levels. FOXF1/FOXF2 directly repressed *CDKN1A* transcription, and the silencing of *CDKN1A* rescued proliferation in FOXF1/FOXF2-deficient cells. Combined depletion completely blocked tumor growth, while overexpression enhanced it [109].

In FP-RMS, *FOXF1* is highly expressed and directly activated by the *PAX3-FOXO1* oncogene via enhancer binding. CRISPR/Cas9-mediated inactivation of FOXF1 or its enhancers impaired tumor growth, even in the presence of *PAX3-FOXO1*. Loss of FOXF1 promoted myogenic differentiation in FP-RMS cells, while its overexpression suppressed differentiation in normal myoblasts. FOXF1 also cooperates with *PAX3-FOXO1*, MYOD1, and MYOG to regulate the FP-RMS transcriptional network [110].

**Figure 2:**
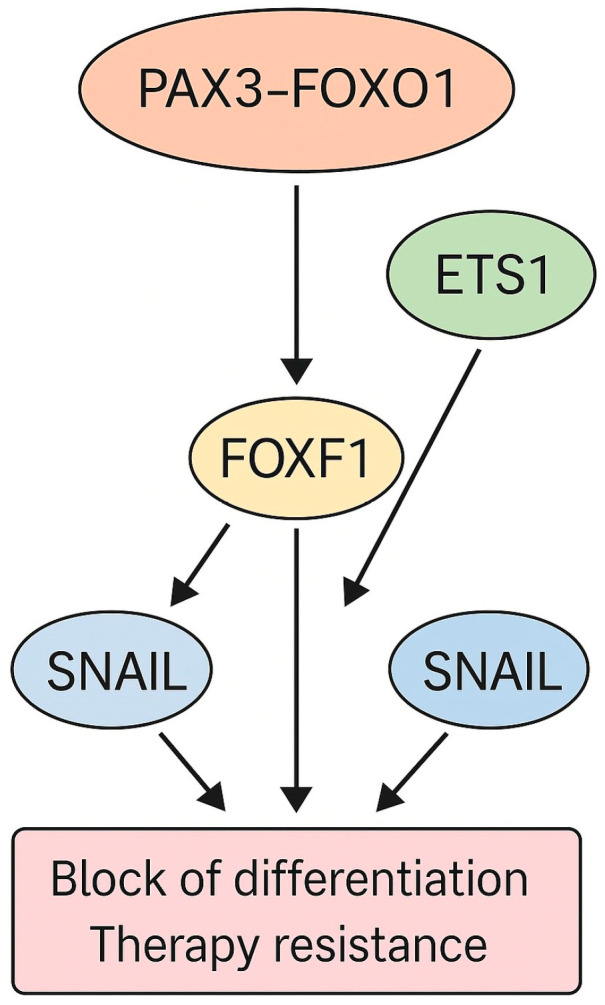
Functional interplay between *PAX3-FOXO1*, FOXF1, ETS1, and SNAIL in promoting dedifferentiation and therapy resistance in fusion-positive alveolar rhabdomyosarcoma (FP-RMS). The fusion protein *PAX3-FOXO1* transcriptionally activates *FOXF1*, which, in turn, cooperates with ETS1 and SNAIL to repress muscle differentiation and promote therapy resistance in FP-RMS. ETS1 enhances FOXF1 chromatin occupancy and cooperates on shared enhancer elements, while SNAIL contributes to transcriptional repression and mesenchymal-like adaptation. Together, this network forms a differentiation-inhibitory axis downstream of *PAX3-FOXO1*.

Building on this, studies have further elucidated a broader transcriptional axis in which *PAX3-FOXO1* activates secondary regulators that reinforce its oncogenic program. One key effector is *FOXF1*, which, beyond its direct transcriptional induction by *PAX3-FOXO1*, partners with MYOD1 and MYOG to maintain FP-RMS-specific gene expression and block terminal differentiation. Additionally, ETS1 acts as a cofactor of FOXF1, co-occupying enhancer regions and amplifying *PAX3-FOXO1*-driven transcriptional output [111,112]. Finally, SNAIL (encoded by *SNAI1*), a classical regulator of epithelial–mesenchymal transition, represses muscle-specific genes and promotes resistance to both differentiation signals and therapeutic interventions [113]. These interactions are summarized in Figure 2.

This FOXF1-ETS1-SNAIL axis not only reinforces the *PAX3-FOXO1*-driven transcriptional landscape but also actively promotes the key malignant features of FP-RMS. FOXF1 enhances cell proliferation and migration, while ETS1 and SNAIL contribute to epithelial–mesenchymal plasticity and resistance to differentiation-inducing therapies. Together, these transcription factors constitute a therapy-refractory subnetwork downstream of *PAX3-FOXO1* [110,111,112,113].

### 6.2. ETS1 as a Cooperative Transcriptional Effector in PAX3-FOXO1-Driven FP-RMS

ETS1, a member of the E26 transformation-specific (ETS) transcription-factor family, plays a pivotal role in regulating gene-expression programs across diverse biological processes, including cell proliferation, differentiation, apoptosis, and angiogenesis. ETS-family proteins share a conserved winged helix-turn-helix DNA-binding domain (ETS domain), which recognizes GGAA/T-containing motifs known as ETS-binding sites (EBS). Many also contain a Pointed (PNT) domain that mediates protein–protein interactions, allowing transcriptional cooperation with other factors [114,115].

In the context of FP-RMS, ETS1 emerges as a critical co-regulator of oncogenic transcription. It is upregulated downstream of both the *PAX3-FOXO1* fusion oncoprotein and the histone demethylase KDM3A, forming a regulatory axis that converges on key disease-promoting genes [112]. Chromatin mapping studies reveal that ETS1 colocalizes with *PAX3-FOXO1* and KDM3A at enhancers of oncogenic targets such as *FGF8*, *IL4R*, *MEST*, and *PODXL*—the latter being newly identified as a direct effector in FP-RMS progression. Notably, ETS1 physically interacts with *PAX3-FOXO1*, enhancing its chromatin occupancy and transcriptional activity.

Functionally, this cooperative interaction can be disrupted by the small-molecule inhibitor YK-4-279, which interferes with the *PAX3-FOXO1*–ETS1 complex. Treatment with YK-4-279 displaces *PAX3-FOXO1* from chromatin, represses the expression of critical target genes, and significantly reduces tumor-cell proliferation and invasiveness, highlighting its potential as a targeted therapeutic strategy [111,112].

Transcriptomic analyses further support the centrality of the KDM3A–ETS1 axis in FP-RMS pathogenesis. Both factors control large subsets of *PAX3-FOXO1*-induced genes, particularly those under the regulation of super-enhancers, such as MEST. Importantly, ETS1 also regulates a distinct set of FP-RMS-associated genes independently of *PAX3-FOXO1*, suggesting a broader disease-promoting role. Epistatic analysis positions ETS1 functionally downstream of KDM3A and *PAX3-FOXO1*, consolidating its role as an amplifier of oncogenic transcriptional circuits in FP-RMS.

Collectively, these findings define ETS1 not only as a downstream effector of *PAX3-FOXO1* and KDM3A but also as a key modulator of enhancer-driven oncogenic programs. The ETS1-centered transcriptional network represents a potential therapeutic vulnerability in FP-RMS, particularly through pharmacologic disruption of its interaction with *PAX3-FOXO1* [112,114,115,116].

### 6.3. Therapeutic Potential and Limitations of NF-κB Inhibition in Alveolar Rhabdomyosarcoma

The transcription factor NF-κB plays a central role in regulating inflammation, innate immunity, and oncogenesis, and its dysregulation contributes to tumor progression in multiple cancer types, including ARMS [117]. In fusion-positive ARMS, NF-κB activity is aberrantly upregulated, in part due to PI3K/AKT pathway activation, resulting in impaired myogenic differentiation. This effect is mediated by NF-κB-induced activation of cyclin D1/cdk4 complexes, which inhibit MYOD1 function. Accordingly, NF-κB inhibitors—alone or in combination with AKT and/or cdk4 inhibitors—have been proposed to restore differentiation capacity and enhance chemotherapy efficacy in ARMS [118].

At the molecular level, dysregulation of the NF-κB–YY1–miR-29 axis has been shown to suppress the tumor suppressor *miR-29*, contributing to tumorigenesis in ARMS. A large-scale chemical screen across 55 sarcoma models revealed that pharmacologic NF-κB inhibition reduced proliferation in multiple ARMS cell cultures [119]. However, in vivo studies revealed limitations of NF-κB inhibition as a monotherapy. Genetic deletion of IKKβ or pharmacologic blockade of NF-κB did not suppress tumor growth in mouse models and, paradoxically, accelerated tumor onset. Interestingly, tumors lacking NF-κB signaling displayed increased sensitivity to BCL-2 inhibition. Combination treatment with NF-κB and BCL-2 inhibitors, such as navitoclax, showed synergistic anti-tumor activity, suggesting that NF-κB inhibition may be most effective within a rationally designed combination strategy [119].

Moreover, immune escape in ARMS is facilitated by the overexpression of inhibitors of apoptosis proteins (IAPs), which can be targeted with Smac mimetics. While the Smac mimetic BV6 alone did not impair ARMS spheroid growth, pre-treatment with BV6 markedly enhanced NK-cell-mediated cytotoxicity. Mechanistically, BV6 promotes rapid IAP degradation and activates NF-κB signaling, leading to transcriptional reprogramming and the release of caspases, particularly caspase-8, which is essential for the immune response [120]. These findings highlight a dual role of NF-κB in ARMS—as a driver of tumorigenesis and a potential immune sensitizer—underscoring the complexity of targeting this pathway.

In summary, while NF-κB is a key player in ARMS pathogenesis, its inhibition alone may be insufficient for therapeutic benefit. However, in combination with agents such as BCL-2 inhibitors or Smac mimetics, NF-κB modulation may enhance cytotoxicity and overcome therapeutic resistance.

### 6.4. The Role of SNAIL in Alveolar Rhabdomyosarcoma: A Key Regulator of Myogenic Blockade and Therapeutic Target

SNAIL (*SNAI1*), a member of the SNAIL family of zinc finger transcription factors that also includes SLUG (*SNAI2*) and SMUG (*SNAI3*), has emerged as a crucial regulator of myogenesis and a potential therapeutic target in ARMS. SNAIL expression is elevated in various sarcomas, including fibrosarcoma and ARMS, where its levels positively correlate with the tumor stage [121]. The stability of SNAIL is regulated post-translationally by the deubiquitinase USP27X, which prevents its proteasomal degradation [122].

Mechanistically, SNAIL modulates gene expression by binding to E-box motifs (CANNTG) and recruiting histone deacetylases (HDACs), thereby altering the chromatin landscape [122]. In the context of ARMS, SNAIL interferes with normal myogenic differentiation by displacing MYOD from E-box elements, directly blocking its transcriptional activity. Furthermore, SNAIL binds to the promoter of *MYF5*—a critical regulator of early myogenesis—and represses its expression [113]. FP-RMS cells display significantly lower *MYF5* expression compared to ERMS and FN-RMS, likely due to the presence of the *PAX3-FOXO1* fusion protein, which may act upstream of SNAIL in this regulatory cascade [113,123]. Indeed, studies suggest that *PAX3-FOXO1* indirectly contributes to the repression of *MYF5* via the upregulation of SNAIL, reinforcing its role in blocking terminal myogenic differentiation.

SNAIL silencing in FP-RMS cells results in the reactivation of *MYF5* transcription, restoration of MYOD binding to target genes, increased expression of myogenic factors and muscle-specific microRNAs, and ultimately induces differentiation while inhibiting proliferation in vitro. Importantly, in vivo studies have shown that the knockdown of SNAIL abolishes the growth of ARMS xenografts, highlighting its therapeutic potential in differentiation-based treatment strategies [113].

The upstream activation of SNAIL in ARMS is closely linked to the overexpression of transforming growth factor beta 1 (TGF-β1), a pleiotropic cytokine with known roles in cancer progression and immune modulation. Elevated levels of TGF-β1 have been reported in ARMS and lead to the transcriptional activation of SNAIL, thereby promoting epithelial-to-mesenchymal transition (EMT) and further reinforcing the myogenic block [124]. In addition to its effects on differentiation, TGF-β/SNAIL signaling contributes to chemoresistance in ARMS, underscoring its relevance as a molecular driver of tumor aggressiveness.

Collectively, these findings establish SNAIL as a central effector of the differentiation blockade in ARMS and a downstream target of TGF-β signaling. Given its multifaceted role in tumor progression, the inhibition of SNAIL or its upstream activators represents a promising therapeutic avenue to restore myogenic programs and suppress tumor growth in ARMS.

Transcription factors (TFs) are central to the metabolic reprogramming and oncogenic transformation of rhabdomyosarcoma (RMS), particularly in the aggressive alveolar subtype (ARMS). Key TFs such as FOXF1, ETS1, NF-κB, and SNAIL cooperate with oncogenic drivers like *PAX3-FOXO1* and epigenetic regulators to promote tumor growth, block differentiation, and support metastasis. These factors orchestrate complex transcriptional networks and enhancer programs that sustain the malignant phenotype. While each TF presents unique therapeutic opportunities—ranging from direct inhibition to the modulation of upstream signaling pathways—their functional redundancy and pleiotropic effects highlight the necessity of combination strategies. Targeting these TFs or their regulatory axes may offer novel and effective approaches to overcoming the current limitations in RMS treatment and restoring normal differentiation pathways.

## 7. Receptor Tyrosine Kinases RTKs

Tyrosine phosphorylation is a post-translational modification essential for cellular signalization. The enzymes that catalyze phosphoryl transfer to tyrosine residues in protein substrates are protein tyrosine kinases. Of the 90 tyrosine kinases, 58 are receptor types (distributed into 20 subfamilies), and 32 are nonreceptor types (10 subfamilies) [125].

The RTK family includes, among others, epidermal growth factor receptor (EGFR), platelet-derived growth factor receptors (PDGFR), fibroblast growth factor receptors (FGFRs), vascular endothelial growth factor receptors (VEGFR), MET receptor (hepatocyte growth factor/scatter factor [HGF/SF] receptor), Anaplastic lymphoma kinase (ALK), Ephs (ephrin receptors), and the insulin receptor [126]. For clarity, Table 2 presents an overview of actionable RTKs in FP-RMS, their roles in tumor biology, available inhibitors, and the current clinical-trial status.

### 7.1. Anlotinib as a Multi-Target Tyrosine Kinase Inhibitor in FP-RMS

Anlotinib, a multi-target tyrosine kinase inhibitor, acts on receptors such as VEGFRs, PDGFR, and FGFR. The therapy disrupts crucial downstream signaling pathways, effectively thwarting tumor angiogenesis and halting the relentless proliferation of cancer cells. Its anticancer properties have shown promising results across a diverse array of malignancies, including non-small cell lung cancer, colorectal cancer, and breast cancer. Additionally, anlotinib is utilized as a second-line treatment option for soft-tissue sarcomas. This compound not only effectively inhibits the proliferation of ARMS cells but also accelerates apoptosis and induces a significant G2/M phase arrest, significantly impeding tumor growth in vivo. One of the mechanisms behind its anticancer effects is the upregulation of the protein kinase NEK2, which plays a pivotal role in the cellular stress response. This enhances the degradation of *PAX3-FOXO1* through the ubiquitin-proteasome pathway, leading to a marked reduction in *PAX3-FOXO1* protein levels. This distinct action positions anlotinib as one element of a targeted therapeutic strategy for treating *PAX3-FOXO1* fusion-positive rhabdomyosarcoma [132].

### 7.2. FGFR Pathways as Therapeutic Targets in Fusion-Positive Rhabdomyosarcoma

Fibroblast growth factor receptor 4 (FGFR4) is a member of the FGFR receptor tyrosine kinase (RTK) family. It consists of an extracellular ligand-binding domain, a transmembrane helix, and an intracellular tyrosine kinase domain [133]. Upon binding to fibroblast growth factors (FGFs), FGFR4 initiates downstream signaling cascades, including RAS-MAPK, PI3K-AKT, PLCγ, and STAT pathways, which regulate mitogenesis and cellular differentiation. Although FGFR4 plays a vital role in skeletal muscle development and regeneration, it is typically not expressed in differentiated muscle tissues [134,135,136]. The FGFR family contributes to tumorigenesis, angiogenesis, tumor invasion, and drug resistance [135,136,137,138,139].

Both ERMS and ARMS express the *FGFR4* gene, though its protein expression is significantly higher in ARMS and is upregulated early in tumorigenesis. FGFR4 operates downstream of the oncogenic *PAX3-FOXO1* fusion protein [140], and its knockdown compromises ARMS cell viability in vitro [111]. Activating mutations in *FGFR4* occur in up to 7.5% of RMS tumors. These include molecular brake mutations (N535D/K) and gatekeeper mutations (V550E/L), both of which cause constitutive receptor activation and enhance tumor progression and metastatic potential [141]. Importantly, these mutations confer resistance to most type I FGFR inhibitors. Even type II inhibitors, such as ponatinib—currently among the most promising agents in RMS—show limited efficacy against V550E/L mutants [142,143]. Nevertheless, the critical role of *FGFR4* in ARMS supports its designation as a viable therapeutic target [140,141,142,143,144,145].

Beyond mutational activation, *FGFR4* expression can be epigenetically regulated. Guadecitabine (SGI-110), a next-generation DNA methyltransferase inhibitor (DNMTi), reduces H3K4 mono-methylation across the *FGFR4* super-enhancer in ARMS cells, leading to diminished *FGFR4* transcription. This effect correlates with a substantial increase in *KDM5B* mRNA levels—encoding a demethylase that removes H3K4me1 marks [145].

Alternative *FGFR4*-targeting approaches include single-domain antibodies (sdAb) developed by Alijaj et al. [144]. These sdAbs exhibit high specificity and affinity for FGFR4, inhibiting FGF19–FGFR4 signaling via the MAPK pathway. They were also incorporated into targeted liposomal drug delivery systems for FGFR4-positive RMS and used to generate chimeric antigen receptor (CAR) T cells that displayed potent cytotoxicity against FGFR4-expressing tumor cells [144].

Emerging data also underscore the relevance of *FGFR2* in FP-RMS. Cell-based screening using clinically relevant FGFR inhibitors (NVP-BGJ398, nintedanib, dovitinib, ponatinib) revealed higher sensitivity in fusion-positive compared to FN-RMS cell lines, correlating with elevated *FGFR2* and *FGF7* expression levels. The analysis of patient tumor samples confirmed significantly higher mRNA levels of both genes in FP-RMS, suggesting a subtype-specific *FGFR2–FGF7* signaling axis.

Functional studies identified an *FGF7–FGFR2* autocrine loop, marked by sustained MAPK pathway activation and FGF7 secretion under serum starvation. Silencing either *FGFR2* or *FGF7* reduced cell viability. Furthermore, treatment with NVP-BGJ398—an FGFR inhibitor—exhibited synergistic effects with SN38, the active metabolite of irinotecan. In vivo, NVP-BGJ398 significantly suppressed xenograft tumor growth, particularly when combined with irinotecan, underscoring its potential in targeted therapy for FP-RMS [146].

Together, these findings highlight the therapeutic relevance of *FGFR4* and *FGFR2* signaling in fusion-driven rhabdomyosarcoma and support the further development of FGFR-targeted approaches in this malignancy.

### 7.3. PDGFR Signaling in ARMS: A Molecular Target with Context-Dependent Therapeutic Impact

Platelet-derived growth factors (PDGFs) are potent mitogens, chemoattractants, and survival factors for stromal cells such as fibroblasts, smooth muscle cells, and pericytes. These ligands signal through two receptor tyrosine kinases, PDGFRα and PDGFRβ [147,148]. Ligand binding induces receptor dimerization, activation, and autophosphorylation, which, in turn, initiates multiple downstream signaling cascades involving Src, PI3K, PLCγ, and Ras pathways [149,150]. This signaling promotes cellular proliferation, migration, and survival, and is essential during embryogenesis and postnatal development. In adult tissues, PDGF signaling contributes to wound healing, vascular tone regulation, and interstitial fluid pressure homeostasis. Dysregulation of PDGF/PDGFR signaling has been implicated in various proliferative disorders, including ARMS [147,148].

Ehnman et al. demonstrated that PDGFR signaling regulates stemness, differentiation, senescence, and apoptosis in RMS cells. Transcriptomic profiling of 101 primary RMS tumors revealed elevated *PDGF-C* and *PDGF-D* expression across all subtypes. PDGF-CC, PDGF-DD, and PDGFRα were predominantly expressed in tumor cells, whereas PDGFRβ was localized primarily to the vascular stroma [151]. PDGF-CC/PDGFR signaling was associated with developmental gene programs, while PDGF-DD/PDGFRβ activity correlated with pathways involved in wound healing and leukocyte function.

Furthermore, Blandford et al. found that elevated expression levels of PDGFRα and PDGFRβ correlated with reduced failure-free survival and disease progression [152]. In tumors expressing the *PAX3-FOXO1* fusion oncogene and lacking TP53, high levels of PDGF-A, PDGF-C, and PDGFRα were observed. PDGFRα was shown to be a direct transcriptional target of *PAX3-FOXO1* through binding to a homeodomain recognition site in the *PDGFRA* promoter [153].

The therapeutic potential of PDGFR inhibition has been investigated using tyrphostin AG1296, a small-molecule inhibitor of PDGFRα and PDGFRβ. This compound inhibited cell proliferation, viability, and migration, and induced apoptosis in both ARMS and ERMS cells in vitro [154,155]. In ARMS cells, tyrphostin AG1296 decreased AKT expression and ERK phosphorylation [154]. These findings support the rationale for further evaluation of PDGFR inhibition as a therapeutic strategy; however, the translational value of in vitro data remains to be confirmed in in vivo settings.

Functional studies also revealed that PDGFRα signaling supports ARMS cell survival rather than driving cell-cycle progression. Knockdown of *PDGFRA* led to increased tumor-cell apoptosis. In vivo, inhibition using imatinib or PDGFRα-neutralizing antibodies significantly reduced tumor growth, particularly in larger lesions. However, smaller tumors and metastases demonstrated therapeutic resistance, suggesting context-specific limitations and the need for combinatorial or alternative strategies [156].

### 7.4. MET Receptor in ARMS: A Key Regulator of Metastatic Behavior and Differentiation

The MET receptor tyrosine kinase plays a crucial role during embryogenesis and remains functionally active in adult tissues. Upon binding to its physiological ligand, hepatocyte growth factor (HGF), MET triggers signaling pathways that regulate cellular proliferation, differentiation, and motility. The dysregulation of MET signaling, through either overexpression or constitutive activation, has been implicated in tumor progression across various cancer types. Therapeutic strategies targeting MET often involve monoclonal antibodies or small-molecule inhibitors aimed at suppressing these aberrant signaling cascades [157].

In the context of rhabdomyosarcoma (RMS), MET signaling appears to be particularly relevant for the alveolar subtype (ARMS). Compared to ERMS, MET protein expression is significantly elevated in ARMS and is associated with poorer clinical outcomes. However, inter-patient variability in MET expression has been observed. Notably, Chen et al. reported higher MET levels in patients with stage IV disease, fatal outcomes, and, in cases of fusion-positive ARMS (FP-RMS), supporting the notion that MET is transcriptionally regulated downstream of the *PAX3-FOXO1* fusion oncogene [34,89]. Importantly, this overexpression is not attributed to mutations in exons 14–21 of the *MET* gene or to gene amplification. Instead, *MET* mRNA and protein levels are concordant in high-expressing cells, and MET remains constitutively active.

Aberrant MET signaling disrupts normal myogenic differentiation while enhancing cell proliferation and suppressing apoptosis. Introduction of the *TPR-MET* fusion oncogene into ERMS cells, where *TPR* encodes a leucine zipper motif fused to the MET kinase domain [158], resulted in increased metastatic potential and enhanced vascularization. These changes were linked to the upregulation of VEGF, MMP9, and *miR-378* expression. Furthermore, the forced differentiation of ARMS cells led to a reduction in MET expression, while MET silencing conversely promoted myogenic differentiation. This was marked by a morphological shift toward spindle-shaped cells characteristic of muscle fibers [159].

The therapeutic inhibition of MET reduced the expression of the early myogenic marker MyoD and attenuated metastatic potential, particularly to bone marrow. This was accompanied by a decrease in CXCR4 expression and impaired cellular migration in response to gradients of HGF and SDF-1/CXCL12 [160,161,162]. These findings highlight the dual role of MET in maintaining the undifferentiated, aggressive phenotype of ARMS cells and facilitating metastasis.

Altogether, targeting MET expression or function may offer therapeutic benefit by limiting ARMS aggressiveness and promoting terminal myogenic differentiation, positioning MET as a viable candidate for differentiation-based or anti-metastatic treatment strategies in fusion-positive ARMS.

### 7.5. Mechanisms of Resistance to RTK Inhibition in FP-RMS

Despite promising preclinical data, the therapeutic targeting of RTKs in fusion-positive rhabdomyosarcoma has yielded limited clinical success. A major contributing factor is receptor crosstalk—where inhibition of one RTK (e.g., IGF1R) results in the compensatory activation of parallel pathways such as MET, EGFR, or PDGFR, thereby sustaining downstream signaling (e.g., PI3K/AKT, MAPK) [163]. Additionally, ligand redundancy and autocrine loops can restore receptor activity despite kinase inhibition, as observed for IGF1R and PDGFRβ in RMS cell lines [151,164].

Another critical mechanism is the adaptive reprogramming of transcriptional networks. Upon RTK blockade, *PAX3-FOXO1*-positive cells may switch dependency to other survival pathways, a phenomenon enhanced by epigenetic plasticity [165]. These observations underscore the need for combination therapies that simultaneously target RTKs and compensatory signaling or epigenetic regulators to overcome intrinsic and acquired resistance.

### 7.6. TRIB3 in ARMS: A Promising Pseudokinase Target Amidst Therapeutic Challenges

Pseudokinases are a distinct and increasingly recognized subgroup within the kinase superfamily, characterized by their lack of classical catalytic activity. Instead, they engage in non-enzymatic signaling through protein–protein interactions or scaffold functions. Despite their regulatory roles in critical signaling pathways, the therapeutic potential of pseudokinases remains largely underexplored due to the inherent challenges in directly modulating their functions. While conventional kinases have been extensively targeted with small-molecule inhibitors, there is a notable paucity of compounds designed to selectively inhibit pseudokinases, highlighting a promising, yet untapped, area of drug discovery [166].

Tribbles homolog 3 (TRIB3) is one such pseudokinase with significant implications in cancer biology. As a member of the TRIB protein subfamily, which also includes TRIB1 and TRIB2, TRIB3 shares a conserved pseudokinase domain, structurally resembling active kinases but lacking enzymatic function. TRIB3 has emerged as a critical regulator of various malignancies, in part due to its ability to modulate the activity of transcription factors such as FOXO1 [166,167].

In ARMS, TRIB3 expression is markedly elevated and has been specifically linked to the presence of the *PAX3-FOXO1* fusion oncoprotein, a defining molecular hallmark of this aggressive tumor subtype. Functional studies using the genetic inhibition of TRIB3 revealed a significant decrease in cell proliferation, implicating TRIB3 as a driver of oncogenic growth in ARMS. Notably, TRIB3 knockdown not only reduced *PAX3-FOXO1* protein levels but also downregulated the expression of its downstream target genes. This was accompanied by the suppression of the Akt signaling cascade, a major pathway associated with tumor-cell survival and proliferation [168].

**Figure 3:**
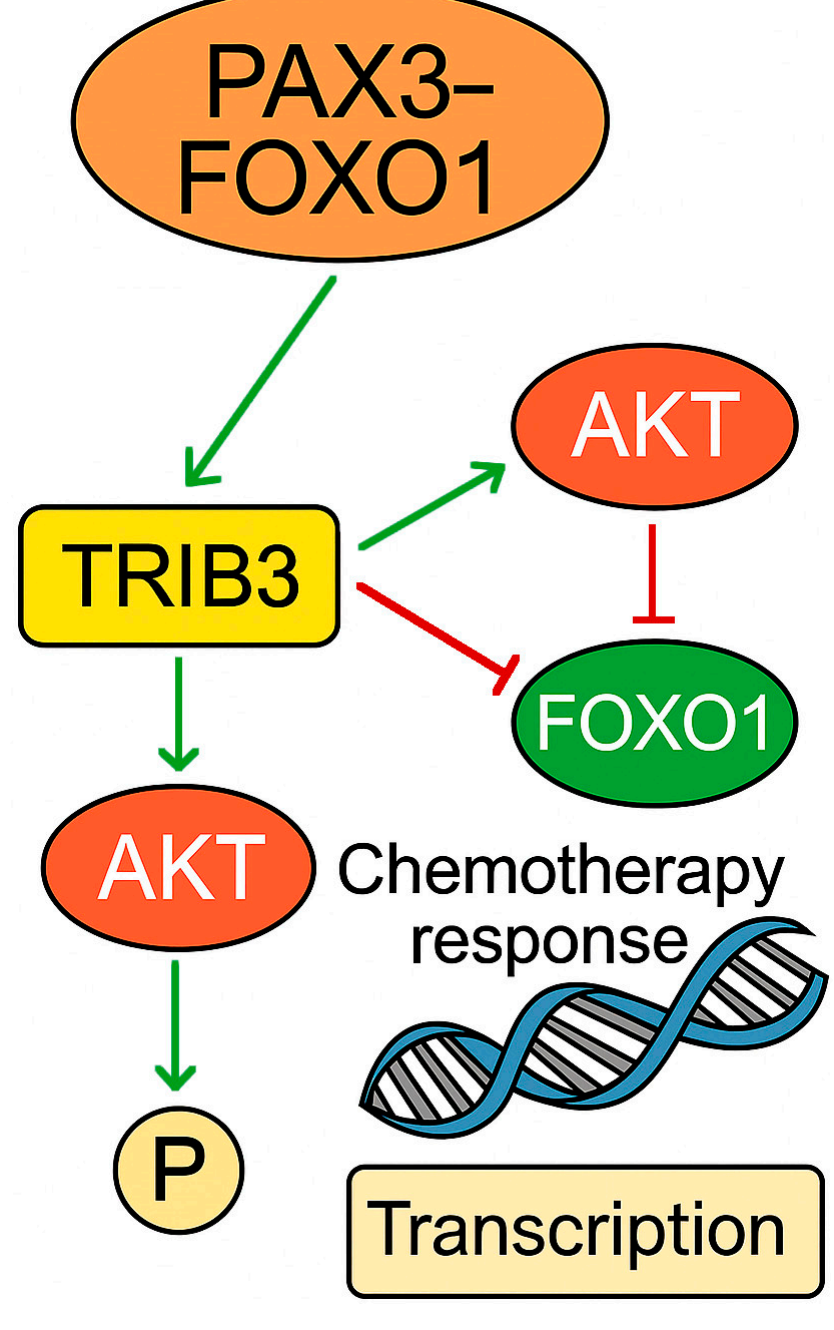
TRIB3 integrates *PAX3-FOXO1* activity with AKT signaling and FOXO1 suppression in high-risk rhabdomyosarcoma. This schematic illustrates the regulatory network involving TRIB3 in fusion-positive rhabdomyosarcoma (FP-RMS). The fusion oncogene *PAX3-FOXO1* transcriptionally upregulates TRIB3, which, in turn, sustains AKT-pathway activation and promotes phosphorylation-mediated survival signals. TRIB3 also reinforces the stability or expression of *PAX3-FOXO1*, forming a positive feedback loop. Additionally, TRIB3 suppresses the FOXO1 transcription factor, which would otherwise promote stress responses, apoptosis, and chemosensitivity. Through these interactions, TRIB3 shifts the balance away from differentiation and apoptosis toward oncogenic maintenance, transcriptional reprogramming, and chemotherapy resistance in FP-RMS [Based on [168,169,173]]. Green arrows—activation; red arrows—inhibition.

Moreover, the inducible silencing of TRIB3 in vivo led to a substantial delay in tumor progression and was associated with prolonged overall survival, underscoring the therapeutic relevance of targeting this molecule. Emerging evidence also points to TRIB3 as a contributor to chemoresistance in rhabdomyosarcoma, suggesting a broader role in therapeutic evasion and disease recurrence [169]. A schematic summary of TRIB3’s interactions with the *PAX3-FOXO1*–TRIB3–AKT axis and its role in modulating chemotherapy response is illustrated in Figure 3.

TRIB3 is not specific to alveolar rhabdomyosarcoma (ARMS); rather, it is a stress-response gene expressed across multiple sarcoma subtypes. While TRIB3 expression is particularly elevated in fusion-positive ARMS—especially in tumors harboring the *PAX3-FOXO1* fusion—it is also detectable at lower levels in fusion-negative RMS and is upregulated in other pediatric and adult sarcomas, including Ewing sarcoma, osteosarcoma, and myxoid liposarcoma. In these contexts, TRIB3 is induced by oncogenic stress pathways such as the unfolded protein response and ATF4 signaling, and contributes to cell survival through the modulation of AKT signaling. For example, TRIB3 is a direct ATF4 target in Ewing sarcoma [170] and is elevated in FP-RMS tumors relative to normal tissue and fusion-negative RMS [171]. It also appears among the top hub genes in osteosarcoma diagnostic models and has been associated with prognostic significance in TCGA sarcoma datasets [172]. Thus, TRIB3 appears to function as a broadly activated component of sarcoma stress adaptation rather than as a marker unique to FP-RMS.

Collectively, these findings position TRIB3 as a promising candidate for targeted therapy in ARMS. Its pseudokinase nature, interaction with key oncogenic drivers such as *PAX3-FOXO1*, and involvement in resistance mechanisms make it a compelling focus for future research aimed at developing novel treatment strategies for high-risk RMS patients.

Targeting critical molecular components involved in FP-RMS biology offers a promising avenue for therapeutic advancement. Within this context, several classes of kinases, including both enzymatically active receptor tyrosine kinases (RTKs) and catalytically inactive pseudokinases, have emerged as attractive candidates.

Pseudokinases such as TRIB3, despite lacking classical kinase activity, play pivotal roles in regulating oncogenic transcription factors and survival pathways. Additionally, active RTKs such as FGFR4, FGFR2, PDGFRα/β, VEGFR, and MET have shown subtype-specific overexpression or mutation in FP-RMS, and preclinical data highlight the efficacy of both small-molecule inhibitors and biologics in disrupting these signaling axes. Notably, multi-target TKIs like anlotinib and FGFR-specific inhibitors have demonstrated synergistic potential when combined with conventional chemotherapy, offering a rationale for integrative treatment approaches.

While this section has focused on selected RTKs and pseudokinases with substantial preclinical validation, further receptor tyrosine kinases implicated in FP-RMS—including ALK, IGF1R, EphB4, and EGFR—are discussed in more detail in Section 10. Together, these findings underscore the urgent need for the continued exploration of kinase-targeted strategies to overcome therapeutic resistance and improve clinical outcomes for patients with FP-RMS.

## 8. Disarming ARMS: Cytokine Crosstalk, Autoantibodies, and Immune-Effector Engineering in Alveolar Rhabdomyosarcoma

Immunotherapy, which harnesses components of the immune system to recognize and eliminate malignant cells, has transformed the therapeutic landscape of several cancer types. Its modalities range from immune checkpoint inhibitors to cellular therapies and antibody-based interventions, offering the potential for durable responses, even in advanced disease stages [174].

Despite these advances, the application of immunotherapy in solid tumors, such as ARMS, remains limited. ARMS is resistant to immune-mediated clearance due to its immunosuppressive microenvironment and molecular heterogeneity [175,176]. Nonetheless, recent research has uncovered promising immunologic vulnerabilities in ARMS, including cytokine-driven signaling pathways, tumor-specific autoantibody responses, and surface antigen expression amenable to immune effector targeting.

### 8.1. Targeting Cytokine Signaling in ARMS: The Dual Role of IL-4R and IL-24

Recent studies have highlighted the importance of cytokine-mediated immune regulation in the pathogenesis and progression of ARMS. In particular, signaling through the interleukin-4 receptor alpha (IL-4Rα) has emerged as a critical modulator of tumor cell behavior. IL-4Rα is broadly expressed on innate and adaptive immune cells, where it transduces signals from type 2 cytokines such as interleukin-4 (IL-4) and interleukin-13 (IL-13). IL-4 is a pleiotropic cytokine produced by a range of immune and non-immune cells, including basophils, mast cells, eosinophils, neutrophils, NKT cells, and keratinocytes [177,178]. It plays an essential role in immune regulation and also influences myogenic differentiation.

Hosoyama et al. [179] investigated the function of IL-4R-mediated signaling in RMS and demonstrated that ARMS cells exhibit significantly elevated IL-4R expression compared to normal skeletal muscle [33]. In both human RMS cell lines and primary murine ARMS cultures, stimulation with IL-4 and IL-13 promoted cell proliferation and activated downstream signaling via STAT6, Akt, and MAPK pathways. Simultaneously, IL-4/IL-13 exposure suppressed myogenic differentiation by downregulating *MYOD* and *MYOG* expression. In vivo, the blockade of IL-4R signaling significantly reduced both lymphatic and hematogenous metastasis, leading to prolonged survival in ARMS models [179]. These findings suggest that IL-4R may serve as a potential immunotherapeutic target, particularly in combination with standard multimodal therapies.

In contrast, interleukin-24 (IL-24), also known as melanoma differentiation-associated gene 7 (mda-7), functions as a tumor-suppressor cytokine. A member of the IL-10 family, IL-24 modulates the expression of multiple genes and pathways involved in apoptosis, the inhibition of proliferation and migration, and the reversal of drug resistance [180,181,182,183]. In ARMS, IL-24 overexpression leads to decreased tumor cell survival, growth, and motility. Notably, IL-24 expression is inversely correlated with *PAX3-FOXO1*, a fusion oncogene characteristic of ARMS. Mechanistically, *PAX3-FOXO1* represses IL-24 expression in a histone deacetylase 5 (HDAC5)-dependent manner [29,184,185]. The knockdown of *PAX3-FOXO1* results in decreased HDAC5 expression and subsequent induction of IL-24 [186]. Furthermore, Lacey et al. [186] demonstrated that silencing IL-24 reverses the anti-proliferative and anti-migratory effects observed after *PAX3-FOXO1* knockdown, suggesting that part of *PAX3-FOXO1*’s oncogenic function is mediated through the suppression of IL-24.

Importantly, IL-24 also showed therapeutic potential in vivo, where adenoviral delivery significantly inhibited ARMS tumor growth. These promising results support IL-24 as a candidate for immunotherapeutic intervention. Notably, IL-24-based gene therapy has already demonstrated safety and biological activity in Phase I clinical trials involving patients with advanced solid tumors [186].

Together, these findings underscore the complex and context-dependent role of cytokines in ARMS pathobiology. While IL-4R signaling promotes tumor progression and immune evasion, IL-24 acts as a natural brake on tumor growth. Modulating these pathways may offer new avenues for targeted immunotherapy in ARMS.

### 8.2. Autoantibody Signatures in ARMS: Diagnostic and Therapeutic Implications

During tumorigenesis, cellular transformation alters protein-expression patterns, triggering immune recognition and a humoral response. Although tumor antigens may remain undetectable in early stages, the antibody amplification response enables the identification of tumor-associated autoantibodies as sensitive biomarkers. Advances in proteomics and immuno-proteomics now allow for the discovery of tumor-specific neo-epitopes, offering insights into cancer biology and the potential for autoantibody-based diagnostics and therapies [187].

ARMS induces a tumor-specific autoantibody response. To characterize this response, human protein microarrays were probed with plasma from ARMS patients and healthy controls, revealing 48 differentially reactive tumor-associated antigens (TAAs).

Two candidates—*GTF2i* and *PCDHGC5*—were validated in an extended cohort. Both showed tumor-restricted overexpression, and corresponding autoantibodies effectively distinguished ARMS patients from controls and other sarcoma subtypes. Low anti-PCDHGC5 antibody levels were associated with poorer event-free survival and proved to be an independent negative prognostic factor [188].

These findings provide the first autoantibody profile of ARMS and highlight GTF2i and PCDHGC5 as potential targets for antibody-based diagnostics and immunotherapies.

### 8.3. Immunotherapy Strategies Targeting ERBB2 in Alveolar Rhabdomyosarcoma

While chimeric antigen receptor (CAR), T-cell therapy has revolutionized treatment outcomes for refractory B-cell malignancies, but its efficacy in solid tumors, including ARMS, remains limited. This is largely due to the immunosuppressive tumor microenvironment, poor T-cell infiltration, and suboptimal T-cell function [175,176]. To overcome these barriers, alternative immune effector cells, such as cytokine-induced killer (CIK) cells and natural killer (NK) cells, have been investigated.

CIK cells are a heterogeneous population of ex vivo-expanded, polyclonal T cells that acquire NK-like cytotoxicity during culture, resulting in a distinctive T-NK phenotype. Notably, they exhibit minimal alloreactivity, enabling the use of haploidentical donors in adoptive therapies. Merker et al. [189] assessed the therapeutic efficacy of *ERBB2*-directed CAR-modified CIK cells in a xenograft model of disseminated high-risk rhabdomyosarcoma. In untreated mice, tumors rapidly engrafted following an intravenous tumor cell injection, resulting in early mortality. Wild-type CIK cells, administered during the early disease stages, delayed tumor progression in 67% of mice, while *ERBB2*-CAR CIK cells prevented tumor engraftment in all treated animals. Compared to unmodified CIKs, CAR-engineered counterparts exhibited improved tumor control and were associated with selective accumulation of NK and T-NK subpopulations at tumor sites, suggesting the enhanced engagement of innate immune mechanisms. However, in cases of advanced disease, CIK-based therapies only delayed progression, underscoring the importance of early therapeutic intervention.

Heim et al. [190] provided complementary evidence using NK-92/5.28.z cells—an *ERBB2*-targeted, CAR-engineered NK cell line. These cells efficiently lysed *ERBB2*-positive RMS cells in vitro and significantly suppressed tumor progression in a metastatic xenograft model. Moreover, NK-92/5.28.z cells persisted within tumor sites, further supporting their potential as off-the-shelf cellular immunotherapies for metastatic RMS.

Collectively, these findings underscore the potential of CAR-modified CIK and NK cell therapies as promising alternatives to conventional CAR-T approaches for ARMS, particularly when targeting *ERBB2*. By leveraging innate immune cytotoxicity and reducing alloreactivity, these strategies may offer improved efficacy in the solid-tumor setting and merit further clinical exploration.

For an expanded discussion on the role of tyrosine kinase receptors, including *ERBB2*, in rhabdomyosarcoma pathogenesis, see De Giovanni et al. [191].

Immunotherapy is emerging as a promising avenue in the treatment of ARMS, offering potential where conventional therapies often fall short. Recent advances highlight the complex interplay between tumor-promoting and tumor-suppressing immune signals—such as *IL-4Rα*-driven progression and *IL-24*-mediated inhibition—as well as novel targets like ERBB2 and tumor-associated autoantigens. These findings underscore the importance of immune-based diagnostics and precision therapies. Future strategies should focus on early intervention, combination regimens, and optimizing immune-effector-cell delivery. Continued translational research and clinical trials will be essential to bring these immunotherapeutic approaches into routine care for ARMS patients.

## 9. Epigenetic Mechanisms in the Pathogenesis and Therapeutic Targeting of Alveolar Rhabdomyosarcoma

Recent advances in cancer biology have highlighted the crucial role of epigenetic mechanisms—such as DNA methylation, histone modifications, and chromatin remodeling—in the initiation and progression of pediatric malignancies, including rhabdomyosarcoma (RMS) [112,116,192,193]. Unlike genetic mutations, epigenetic alterations are reversible and dynamically regulated, offering a unique layer of transcriptional control that is particularly relevant during development and tissue differentiation. These characteristics make them attractive candidates for the targeted therapeutic intervention.

In ARMS, epigenetic dysregulation plays a pivotal role in disease biology. A defining feature of ARMS is the presence of *PAX3-FOXO1*, which reprograms the epigenetic landscape to sustain tumor growth. Through its interaction with chromatin-modifying complexes and transcriptional coactivators, *PAX3-FOXO1* establishes aberrant super-enhancers and drives the expression of genes essential for tumor proliferation and survival [194]. This epigenetic rewiring not only underpins tumor initiation but also contributes to therapy resistance and poor clinical outcomes.

The functional dependency of ARMS cells on these epigenetic regulators introduces novel therapeutic vulnerabilities. Inhibitors targeting bromodomain proteins (such as BRD4), histone deacetylases (HDACs), and DNA methylation machinery have shown preclinical efficacy in reversing oncogenic transcriptional programs. Despite these promising developments, the complexity and context-dependence of epigenetic regulation in ARMS demand further mechanistic insights to guide the development of effective epigenetic therapies [112,116,192,193].

This section outlines the current understanding of epigenetic regulation in ARMS, with a focus on its mechanistic role in tumorigenesis and its potential as a targetable axis in future therapeutic strategies.

### 9.1. DNA Methylation in Rhabdomyosarcoma: Mechanistic Insights and Therapeutic Potential

DNA methylation is a fundamental epigenetic mechanism involving the addition of a methyl group to the C5 position of cytosine, resulting in 5-methylcytosine. This modification regulates gene expression by recruiting repressive protein complexes or inhibiting transcription factor binding. During development, DNA methylation patterns undergo dynamic changes through coordinated de novo methylation and demethylation, ultimately establishing stable, cell type-specific epigenetic profiles. These patterns are crucial for controlling tissue-specific gene expression [195].

DNA methylation represents a complex and challenging area in cancer research. Methylation profiling of RMS samples has revealed distinct patterns among metastatic FP-RMS, localized FN-RMS, and ERMS subtypes [196]. These epigenetic differences underscore the potential role of DNA methylation in tumor heterogeneity, progression, and metastatic behavior. Given the reversible and dynamic nature of epigenetic modifications, targeted epigenetic therapy has emerged as a promising strategy for RMS treatment. As emphasized by Tombolan et al. [197], DNA methylation is a particularly compelling therapeutic target, as it can be pharmacologically modulated without altering the underlying DNA sequence. This offers the potential to reactivate silenced tumor-suppressor genes or disrupt oncogenic pathways.

Sun et al. [198] demonstrated that epigenetic alterations in RMS enhance molecular classification by interacting with genetic mutations and transcriptional programs. DNA methylation profiling identified distinct subtypes associated with fusion status in FP-RMS and RAS mutations in FN-RMS. Notably, the methylation profile of normal muscle tissue closely resembled that of FN-RMS with wild-type RAS. FP-RMS tumors displayed promoter and intergenic hypomethylation along with 3’ UTR hypermethylation. *PAX3-FOXO1* binding sites were enriched among highly overexpressed genes with hypomethylated promoters.

In a follow-up study, Sun et al. [199] analyzed DNA methylation patterns in genetically engineered mouse models (GEMMs) of RMS. These models revealed clusters corresponding to human FP and FN subtypes based on *PAX3-FOXO1* expression. Two epigenetically distinct FN-like subtypes emerged, linked to Pax7 and Myf5 cellular lineages. Integrative analysis across human and mouse models identified shared differentially methylated and expressed genes, highlighting the combined roles of driver mutations and cell-of-origin in shaping the RMS epigenetic landscape.

Further genome analysis indicated hypermethylation of the *PCDHA4* protocadherin gene cluster in metastatic cells, improving the molecular classification of RMS. The *PCDHA4* gene encodes protocadherin alpha 4, a calcium-dependent cell-adhesion protein involved in cellular self-recognition and discrimination [200]. The silencing of *PCDHA4* was associated with increased cell proliferation and invasiveness. These findings suggest that *PCDHA4* functions as a tumor suppressor in RMS and that its expression is likely repressed via DNA methylation, particularly in the FP-RMS subgroup. In vitro treatment with the demethylating agent 5-Aza-dC and the histone deacetylase inhibitor TSA restored *PCDHA4* expression. This supports the existence of epigenetic regulation of the gene and demonstrates the potential utility of combination epigenetic therapies [197].

DNA methylation plays a key role in RMS development and subtype classification by regulating gene expression and interacting with genetic mutations. Distinct methylation patterns are associated with tumor subtypes and metastatic potential. Targeting these reversible epigenetic changes, such as *PCDHA4* silencing, offers promising therapeutic opportunities.

### 9.2. Chromatin Remodeling and Epigenetic Reprogramming in Fusion-Positive Rhabdomyosarcoma

Dynamic changes in chromatin conformation reshape genome organization and are pivotal in regulating gene transcription. Chromatin structure is modulated through reversible, enzyme-catalyzed covalent modifications and non-covalent, ATP-dependent remodeling by complexes such as switch/sucrose nonfermentable (SWI/SNF), inositol-requiring 80 (INO80), imitation switch (ISWI), and chromodomain-helicase DNA-binding protein (CHD). Emerging evidence underscores the crucial role of chromatin remodeling during various stages of postnatal and adult neurogenesis. Conversely, its dysregulation can impair epigenetic gene regulation and promote pathological transcriptional programs, contributing to disease development [201].

Epigenetic regulation has emerged as a key axis of transcriptional control in FP-RMS. The chromatin remodeler CHD4, a core ATPase subunit of the nucleosome remodeling and deacetylase (NuRD) complex, has been identified as an essential dependency in FP-RMS. A NuRD-targeted CRISPR-Cas9 screen revealed that FP-RMS cells are uniquely sensitive to CHD4 depletion compared to other NuRD components, indicating a non-redundant, lineage-specific role for CHD4 in this tumor type [202].

Mechanistically, CHD4 is recruited to super-enhancer regions where it facilitates chromatin remodeling to permit *PAX3-FOXO1* binding—an interaction essential for activating the oncogenic transcriptional program. Loss of CHD4 disrupts this process by evicting histone deacetylase 2 (HDAC2) from chromatin, which leads to increased histone acetylation, impaired RNA Polymerase II recruitment, and defective transcriptional initiation [202,203].

Further highlighting its role, Böhm et al. [204] showed that CHD4 directly interacts with *PAX3-FOXO1* via short DNA fragments and co-occupies regulatory elements of key target genes. CHD4 is required for the expression of a specific subset of *PAX3-FOXO1* target genes. Its loss reduced FP-RMS cell viability in vitro and mimicked the effects of *PAX3-FOXO1* depletion. In vivo, CHD4 knockdown induced regression of FP-RMS xenograft tumors, reinforcing its therapeutic potential.

In addition, integrative analyses of genome-wide cancer dependency datasets have identified CHD4 as a broader oncogenic vulnerability, suggesting that its role in maintaining chromatin architecture and transcriptional output may be essential across multiple tumor types [202].

Recent studies have also revealed that the *PAX3-FOXO1* fusion transcription factor possesses pioneer factor activity, enabling it to bind inaccessible chromatin regions by recognizing partial or degenerate homeobox motifs [205]. This property allows *PAX3-FOXO1* to initiate chromatin remodeling during early phases of gene activation. Upon binding to loci containing composite paired-box/homeobox motifs, *PAX3-FOXO1* promotes local chromatin opening and facilitates the redistribution of the active histone mark H3K27ac, increasing chromatin accessibility. These changes are associated with the activation of neural lineage transcriptional programs [194].

Importantly, the neural gene expression profiles induced by *PAX3-FOXO1* closely match those observed in clinical rhabdomyosarcoma samples, particularly post-chemotherapy, suggesting a role in therapy resistance [206]. Collectively, these findings establish *PAX3-FOXO1* as both a chromatin pioneer and a master regulator of neural gene activation, emphasizing its central role in FP-RMS initiation, maintenance, and progression [194].

Moreover, *PAX3-FOXO1* reprograms the epigenetic landscape of FP-RMS by generating novel super-enhancers (SEs). These SEs, formed in cooperation with myogenic transcription factors such as MYOG, MYOD, and MYCN, establish a conserved autoregulatory circuit across cell lines and primary tumors. This enhancer network confers a unique vulnerability: FP-RMS cells are particularly sensitive to small-molecule inhibitors targeting proteins bound at *PAX3-FOXO1*-associated enhancers. Notably, *PAX3-FOXO1* recruits and depends on the BET protein BRD4 to maintain this network, making BRD4 inhibition a promising therapeutic strategy [39].

CHD4 and *PAX3-FOXO1* cooperatively remodel chromatin to drive oncogenic programs in FP-RMS. Their essential, lineage-specific roles make them attractive therapeutic targets, with BRD4 inhibition showing particular promise for disrupting this epigenetic network.

Recent studies have demonstrated the therapeutic relevance of targeting epigenetic regulators in FP-RMS, including EZH2, KDM3A, and KDM4B. Among the emerging small-molecule inhibitors, QC6352 has shown strong preclinical activity and is characterized as a highly selective pan-KDM4 inhibitor with minimal off-target toxicity in vivo [207]. It exhibits on-target effects in FP-RMS models, phenocopying genetic *KDM4B* knockout and synergizing with standard chemotherapy [208]. Although QC6352 itself remains in preclinical development, the structurally related inhibitor TACH101 has entered a Phase I clinical trial for advanced solid tumors (ClinicalTrials.gov ID: NCT05076552) [209], validating the clinical feasibility of KDM4 inhibition.

In contrast, P3FI-90 (also referred to as P3F-90) is a dual KDM3B and LSD1 inhibitor identified through a functional screen for compounds suppressing *PAX3-FOXO1* activity [210]. While less selective than QC6352, P3FI-90 displays specificity toward lysine demethylases and lacks significant activity against unrelated epigenetic enzymes. In vivo, it delays tumor growth in FP-RMS xenograft models and modulates enhancer histone marks, recapitulating the effects of KDM3B or LSD1 knockdown. However, it is currently at the lead optimization stage and has not yet entered clinical testing.

Together, these findings support the ongoing development of selective and combinable epigenetic therapies for FP-RMS, while highlighting the importance of compound selectivity and translational readiness in evaluating therapeutic viability.

### 9.3. Epigenetic Regulation via Histone Modifications in Alveolar Rhabdomyosarcoma

Histone modifications are controlled by chromatin-modifying enzymes that read specific positions and add or remove appropriate covalent modifications. The repertoire of histone modifications continues to expand, and the intricacies of their functional mechanisms are only beginning to be elucidated. Nevertheless, it is increasingly evident that these modifications play pivotal roles in a wide array of biological processes, particularly those governing the regulation and expression of DNA [211].

#### 9.3.1. BMI1 and Polycomb-Mediated Histone Modifications in ARMS

BMI1 (B lymphoma Mo-MLV insertion region 1), also known as polycomb group factor 4 (PCGF4), has emerged as a pivotal epigenetic regulator implicated in the maintenance of aggressive cancer phenotypes, including ARMS. BMI1 is a core component of the Polycomb Repressive Complex 1 (PRC1) [212], one of two primary polycomb group complexes involved in transcriptional repression through chromatin remodeling.

Polycomb group proteins—including PRC1 and PRC2—modulate critical developmental and cellular programs such as pluripotency, stem-cell self-renewal, and epigenetic memory. They exert their repressive effects via distinct post-translational histone modifications: PRC1 catalyzes the monoubiquitination of histone H2A at lysine 119 (H2AK119ub), while PRC2 mediates the trimethylation of histone H3 at lysine 27 (H3K27me3) [213]. Although BMI1 lacks intrinsic enzymatic activity, it is indispensable for PRC1’s function, reinforcing its role in gene silencing and establishing its oncogenic potential in a variety of cancers [212].

In the context of ARMS, BMI1 expression is consistently elevated across tumor samples, including patient-derived xenografts and various cell line models. Functional studies employing RNA interference (RNAi) or pharmacological inhibition of BMI1 have demonstrated that its depletion leads to significant reductions in cell proliferation and increased apoptosis in vitro. Furthermore, in vivo BMI1 knockdown results in delayed tumor progression, underscoring its importance in tumor maintenance. These findings suggest that BMI1 is a viable and actionable therapeutic target in ARMS, with potential to disrupt key oncogenic epigenetic pathways [214].

#### 9.3.2. Regulation of Histone Lysine Methylation and Demethylation in ARMS

Histone lysine methylation, which occurs on both the tail and globular domains of histone proteins, plays a pivotal role in regulating nearly all DNA-templated processes, including transcription, replication, and DNA repair. This epigenetic modification is dynamic and reversible, coordinated by the activity of lysine methyltransferases (KMTs, “writers”), lysine demethylases (KDMs, “erasers”), and effector proteins (“readers”) that interpret methylation patterns in a context-dependent manner. Tight spatial and temporal control of lysine methylation is essential for proper development, cellular differentiation, and homeostasis. Dysregulation of this system contributes to numerous diseases, including cancer [215].

Among histone methyltransferases, Enhancer of zeste homolog 2 (EZH2, also known as KMT6A), the catalytic subunit of the Polycomb Repressive Complex 2 (PRC2), is one of the most extensively studied. EZH2 catalyzes the trimethylation of histone H3 at lysine 27 (H3K27me3), a repressive chromatin mark associated with gene silencing. In ARMS, particularly in fusion-positive (FP) subtypes, EZH2 is frequently overexpressed. Its depletion triggers apoptosis in FP-RMS cells by derepressing *FBXO32*, a gene crucial for muscle homeostasis. EZH2 silencing reduces H3K27me3 enrichment at the *FBXO32* promoter, thereby activating its expression. The co-depletion of EZH2 and *FBXO32* partially attenuates the pro-apoptotic phenotype, whereas FBXO32 overexpression amplifies apoptosis in EZH2-deficient cells [216]. The pharmacological inhibition of EZH2 using compounds such as 3-Deazaneplanocin A reproduces these effects and suppresses tumor growth in vivo [217].

Moreover, combining EZH2 inhibitors with retinoic acid (RA) enhances myogenic differentiation and reduces proliferation in RMS cell lines more effectively than either treatment alone. RA promotes interferon signaling and apoptosis in FP-RMS, while EZH2 inhibition reactivates myogenic genes in FN-RMS. In both RMS subtypes, EZH2 inhibition leads to the re-expression of genes silenced by H3K27me3 in untreated cells, reflecting epigenetic reprogramming [218].

SUV39H1 (KMT1A), a histone H3 lysine 9 methyltransferase, is responsible for generating H3K9me3, another repressive chromatin mark. In myoblasts, ectopic expression of KMT1A blocks differentiation by repressing myogenic genes and inhibiting MYOD function [219]. MYOD is the myoblast determination protein that acts as a transcriptional activator promoting the transcription of muscle-specific target genes and playing a role in muscle differentiation. Elevated levels of KMT1A in ARMS cells sustain an undifferentiated phenotype. KMT1A knockdown restores MYOD activity, induces expression of differentiation markers, reduces proliferation, and promotes terminal differentiation and tumor regression [220].

Another therapeutic strategy in ARMS involves targeting heme oxygenase-1 (HO-1), an enzyme upregulated in ARMS that promotes proliferation and angiogenesis while suppressing muscle differentiation. HO-1 represses *miR-206*, a microRNA essential for myogenesis, through HDAC4-mediated chromatin remodeling. Inhibiting HO-1 restores *miR-206* levels, activates myogenic gene expression, and reduces tumor growth and vascularization in ARMS models [221,222,223,224].

Euchromatic histone lysine methyltransferase 1 (EHMT1, also known as GLP or KMT1D) catalyzes the dimethylation of H3K9 (H3K9me2), contributing to chromatin compaction and transcriptional repression [225]. EHMT1 is expressed in both primary and relapsed ARMS tumors. Its knockdown impairs cell motility, promotes myogenic differentiation, and reduces tumor burden. Transcriptomic analyses suggest that EHMT1 indirectly controls the expression of the cancer stem cell (CSC) marker *ALDH1A1* by stabilizing the transcription factor C/EBPβ [226]. Given the role of CSCs in therapy resistance and recurrence [227,228], targeting the EHMT1–C/EBPβ–*ALDH1A1* pathway may offer a promising therapeutic strategy in ARMS [226,229].

Euchromatic histone-lysine N-methyltransferase 2 (EHMT2, also called G9a or KMT1C) shares functional similarity with EHMT1 by also generating H3K9me2. In FP-RMS, EHMT2 represses the tumor suppressor gene *PTEN*, enabling the activation of the AKT and RAC1 pathways. The inhibition of EHMT2 reduces proliferation, motility, and tumor growth, effects reversible by RAC1 re-expression [230,231]. Additionally, EHMT2 expression is regulated by DDX5, an RNA helicase that stabilizes *EHMT2* mRNA. Targeting this DDX5–EHMT2–AKT axis decreases viability and tumor progression in FP-RMS xenografts [230].

Lysine demethylases (KDMs) antagonize the action of KMTs by removing methyl groups from histone tails, thereby allowing dynamic chromatin remodeling. Since the discovery of LSD1 (KDM1A), which demethylates H3K4me1/2 and H3K9me1/2, many KDMs have been characterized and linked to cancer pathogenesis [232,233,234]. Notably, Hsp90 inhibitors such as geldanamycin and its analog 17-DMAG act as indirect KDM inhibitors by promoting the degradation of *PAX3-FOXO1* and accumulation of repressive histone marks like H3K9me3 and H3K36me3 at *PAX3-FOXO1* target genes [235,236]. 17-DMAG monotherapy suppresses oncogenic signaling and promotes muscle differentiation in vivo, with synergistic benefits observed when combined with chemotherapy [237].

KDM3A, a Jumonji family member, demethylates H3K9me1/2 and facilitates transcriptional activation. It supports cell proliferation, survival, motility, and resistance to therapy in multiple cancers [192,238]. In ARMS, the depletion of *KDM3A* reduces colony formation, migration, invasion, and tumor growth [116,192]. KDM3A forms a disease-promoting axis with Ets1 and MCAM in both FP- and FN-RMS. MCAM, a surface protein overexpressed in pediatric tumors, mediates invasion and proliferation, while Ets1 regulates expression of chemokines and *PAX3-FOXO1*-dependent genes including MEST, a developmentally regulated gene under super-enhancer control [112,239,240,241]. Suppressing KDM3A, Ets1, or MCAM limits RMS cell growth and invasiveness, identifying the KDM3A/Ets1/MCAM axis as a potential therapeutic target [112,116,242].

KDM3B, closely related to KDM3A, was implicated in RMS by Kim et al., who identified P3FI-90 as a small molecule inhibitor that suppresses *PAX3-FOXO1* transcriptional activity by targeting KDM3B and other KDMs. P3FI-90 increases repressive H3K9me2 levels and activates apoptotic and myogenic pathways without disrupting *PAX3-FOXO1* protein or enhancer binding. In vivo, P3FI-90 delays tumor growth and promotes differentiation [210].

KDM4B, a demethylase acting on H3K9me3/me2 and H3K36me3/me2, was shown by Singh et al. to regulate core transcriptional networks in FP-RMS. The inhibitor QC6352 binds KDM4B, suppresses its activity, delays tumor progression, and exhibits minimal toxicity. When combined with vincristine and irinotecan, QC6352 enhances tumor regression, suggesting broad utility. However, since QC6352 targets multiple KDM4 isoforms, further work is needed to elucidate the specific contributions of individual KDM4 family members [207,208].

Histone lysine methylation and demethylation govern fundamental processes in cellular identity and function, with profound implications in oncogenesis. In alveolar rhabdomyosarcoma, the balance between KMT and KDM activity influences proliferation, differentiation, apoptosis, and therapy resistance. Specific methyltransferases such as EZH2, EHMT1/2, and KMT1A, and demethylases including KDM3A/B and KDM4B, form oncogenic axes that sustain tumor growth and block myogenesis. Pharmacological modulation of these epigenetic regulators, alone or in combination with differentiation agents or chemotherapeutics, holds promise for improving therapeutic outcomes in patients with high-risk ARMS. The continued investigation of these pathways and the development of selective inhibitors may open new frontiers in personalized, epigenetically guided cancer therapy.

#### 9.3.3. Regulation of Histone Acetylation in Fusion-Positive ARMS

Histone acetylation and deacetylation are key epigenetic mechanisms that regulate chromatin structure and gene expression. These processes involve the addition or removal of acetyl groups from lysine residues on histone tails, catalyzed by histone acetyltransferases (HATs, also known as lysine acetyltransferases, KATs) and histone deacetylases (HDACs), respectively. Acetylation neutralizes the positive charge on histones, resulting in chromatin relaxation and transcriptional activation. Conversely, deacetylation increases chromatin compaction and leads to transcriptional repression [243,244]. The dysregulation of these modifications plays a crucial role in the pathogenesis of various cancers, including fusion-positive rhabdomyosarcoma (FP-RMS). In this context, aberrant histone acetylation patterns support the maintenance of oncogenic gene-expression programs driven by the *PAX3-FOXO1* fusion protein [245,246].

Among the KATs, KAT2A (GCN5) and its paralog KAT2B (PCAF) are key regulators of chromatin remodeling, transcription, DNA replication and repair, and cell-cycle progression [247,248,249]. These enzymes preferentially acetylate histone H3 and, to a lesser extent, H4, but also modify non-histone proteins such as MYOD, enhancing its DNA-binding activity [250]. During normal myogenic differentiation, KAT2B promotes MYOD activation. However, in FP-RMS cells, KAT2B shifts its substrate preference and instead acetylates and stabilizes the *PAX3-FOXO1* oncoprotein. KAT2B is markedly overexpressed in FP-RMS tumor samples, and its genetic silencing or pharmacological inhibition reduces *PAX3-FOXO1* protein levels, leading to decreased cell proliferation and impaired tumor growth in xenograft models [251].

Additional evidence for the therapeutic potential of targeting KATs in RMS comes from the study by Tomasiak et al. [252], who evaluated the effects of natural KAT inhibitors garcinol (GAR) and anacardic acid (AA) on RMS cells. Both compounds significantly inhibited cell proliferation, viability, and clonogenicity in vitro. They also induced G2/M cell-cycle arrest, triggered apoptosis, and altered the expression of genes associated with endoplasmic reticulum stress. Notably, GAR and AA sensitized RMS cells to chemotherapeutic agents, supporting their potential use as adjunctive treatments targeting acetylation-dependent mechanisms.

HDACs catalyze the removal of acetyl groups from histone tails, enhancing the positive charge of histones, which strengthens their interaction with DNA and leads to chromatin condensation and transcriptional repression [253]. In humans, eleven zinc-dependent HDAC isoforms are grouped into four classes: Class I (HDACs 1, 2, 3, 8), Class IIa (HDACs 4, 5, 7, 9), Class IIb (HDACs 6, 10), and Class IV (HDAC11) [243]. Class I HDACs are particularly implicated in oncogene activation, tumor progression, and treatment resistance [244]. Dysregulated HDAC activity is a common feature in many cancers, including FP-RMS [245].

Interestingly, in RMS cells, core regulatory transcription factors (CR TFs) are associated with super-enhancers (SEs), which are characterized by high levels of histone acetylation—a hallmark of active gene expression. Paradoxically, these SEs also recruit HDACs, including HDAC1, HDAC2, and HDAC3, suggesting that HDACs may fine-tune the acetylation levels necessary for optimal CR TF function. The inhibition of these HDACs disrupts the 3D chromatin architecture of SEs, impairs CR TF activity, and reduces the transcription of CR TF-regulated genes [245,254].

Metastatic alveolar RMS (ARMS) presents a major clinical challenge, particularly due to chemotherapy resistance driven by the *PAX3-FOXO1* fusion protein, which alters cell-cycle checkpoint control [255]. This resistance can be counteracted by targeting HDAC3, which interferes with a regulatory feedback loop involving SMARCA4, *miR-27a*, and *PAX3-FOXO1*. The inhibition of HDAC3 reduces SMARCA4 activity, leading to the derepression of *miR-27a*. The restored expression of *miR-27a* destabilizes *PAX3-FOXO1* mRNA and re-sensitizes ARMS cells to chemotherapy in both in vitro and in vivo models [256].

A notable therapeutic advancement in FP-RMS has been the application of entinostat (MS-275), a synthetic benzamide derivative and selective inhibitor of class I and IV HDACs. Entinostat exhibits potent anti-tumor activity in both in vitro and in vivo RMS models, particularly when combined with radiotherapy (RT). In RH30 FP-RMS cells, entinostat induces irreversible cytotoxicity, downregulates cyclins A, B, and D1, and upregulates cyclin-dependent kinase inhibitors p21 and p27. Additionally, it suppresses PI3K/Akt/mTOR signaling and N-Myc expression.

Combination therapy with entinostat and RT enhances ROS production, increases DNA damage, impairs clonogenic potential, and induces G2/M cell-cycle arrest. This treatment also downregulates key antioxidant genes, including NRF2, SOD, CAT, and GPx4. In vivo, entinostat significantly inhibits RH30 tumor growth and, when combined with RT, completely abrogates the progression of RT-resistant tumors [257].

Complementary studies using patient-derived xenograft (PDX) models of ARMS confirm the therapeutic relevance of entinostat. In combination with chemotherapeutic agents such as vinorelbine, cyclophosphamide, doxorubicin, and topotecan, entinostat shows substantial efficacy. While entinostat monotherapy led to limited tumor inhibition in two out of three ARMS PDX models, one model responded with near-complete tumor suppression, highlighting inter-tumoral variability in drug responses [246]. Importantly, entinostat has demonstrated favorable tolerability in pediatric clinical trials, further supporting its potential for inclusion in combination therapies for RMS [258].

Histone acetylation and deacetylation play central roles in the epigenetic regulation of FP-RMS, particularly through the modulation of *PAX3-FOXO1* activity and chromatin dynamics. The overexpression of KAT2B and the dysregulation of HDACs, especially HDAC1–3, support oncogenesis and therapy resistance. Pharmacological inhibition of KATs (e.g., with garcinol and AA) and HDACs (e.g., with entinostat) demonstrates promising anti-tumor activity, particularly in combination with standard therapies. These findings underscore the potential of acetylation-targeted epigenetic therapies as a valuable strategy for improving outcomes in FP-RMS.

ARMS involves major epigenetic dysregulation, especially through histone methylation and acetylation. Key enzymes like EZH2, BMI1, KMT1A, and EHMTs repress tumor-suppressor genes and maintain a proliferative, undifferentiated state. Demethylases (e.g., KDM3A/B, KDM4B) reverse these marks and promote tumor growth. Therapeutic inhibition of these enzymes shows promise, especially in combination with chemotherapy or differentiation agents.

In acetylation, the enzyme KAT2B stabilizes the *PAX3-FOXO1* fusion protein, promoting tumor progression. Inhibiting KAT2B or HDACs (notably HDAC1–3) disrupts oncogenic programs and enhances the treatment response.

Overall, targeting histone modifiers offers a potential treatment strategy for high-risk ARMS patients.

### 9.4. RNA-Binding Protein as a Context-Dependent Therapeutic Target in ARMS

RNA-binding proteins (RBPs) play critical roles in post-transcriptional gene regulation, influencing processes such as RNA splicing, stability, localization, and translation. Their dysregulation has been implicated in various cancers, including rhabdomyosarcoma (RMS) [259].

Staufen1 (STAU1) is an RBP that has garnered attention for its involvement in RMS pathogenesis. Studies have demonstrated that STAU1 is significantly upregulated in both ERMS and ARMS tumors and cell lines compared to normal skeletal muscle. Functionally, STAU1 promotes tumorigenesis by enhancing proliferation in ERMS and inhibiting apoptosis in ARMS [260].

Staufen1 is not a classical epigenetic regulator; however, it can interact with various RNA species, including long non-coding RNAs (lncRNAs) and microRNAs (miRNAs), thereby indirectly influencing the epigenetic landscape of cancer cells. Mechanistically, STAU1 influences mitochondrial metabolism in ERMS by stabilizing mRNAs encoding components of oxidative phosphorylation (OXPHOS), thereby supporting tumor-cell proliferation. In ARMS, STAU1 has been shown to regulate autophagy, a process critical for cell survival under stress. The depletion of *STAU1* in ARMS cells leads to reduced autophagic activity and increased apoptosis, effects linked to the destabilization of autophagy-related mRNAs and the suppression of JNK signaling [261,262].

These findings position STAU1 as a context-dependent regulator of survival pathways in RMS and highlight its potential as a therapeutic target. Targeting STAU1 could disrupt tumor-supportive post-transcriptional networks, offering a novel approach for RMS treatment.

### 9.5. MicroRNAs in Alveolar Rhabdomyosarcoma: Functional Roles and Clinical Implications

MicroRNAs (miRNAs) are endogenous, short (~22 nucleotides), non-coding RNAs that regulate gene expression, primarily by binding to complementary sequences in the 3′ untranslated regions (3′ UTRs) of target messenger RNAs, leading to mRNA degradation or translational repression. Despite their small size, miRNAs exert profound regulatory control over various cellular processes, including proliferation, apoptosis, differentiation, and metastasis. In cancer, the dysregulation of specific miRNAs may act either oncogenically or as tumor suppressors, influencing disease progression, therapy resistance, and clinical outcomes [263,264].

In RMS, including ARMS, miRNA dysregulation contributes significantly to tumorigenesis, the maintenance of an undifferentiated state, and metastatic potential. Several muscle-specific miRNAs—such as *miR-206*, *miR-29*, *miR-203*, *miR-335-5p*, *miR-28-3p*, and *miR-193a-5p*—have been shown to play critical roles in disease development and serve as potential prognostic markers or therapeutic targets.

The expression levels of these muscle-enriched miRNAs are markedly reduced in RMS compared to normal skeletal muscle, although they are still higher than in other non-muscle tissues. Among them, *miR-206* has been identified as a key prognostic biomarker: its low expression correlates with poor overall survival and is an independent predictor in metastatic RMS lacking *PAX3/7-FOXO1* fusion genes. Low *miR-206* levels are also associated with higher SIOP staging and the presence of metastases at diagnosis. Functionally, *miR-206* inhibits RMS cell growth and migration, induces apoptosis, and promotes myogenic differentiation. Gene expression analyses link high *miR-206* expression with myogenic pathways, while low levels are associated with MAPK and NF-κB pathway activation [265].

Similarly, miR-29 functions as a tumor suppressor and is repressed in RMS via the NF-κB–YY1–Polycomb axis, a pathway that inhibits differentiation. During normal myogenesis, the downregulation of this axis allows for *miR-29* expression, which accelerates differentiation by targeting YY1. In RMS, the epigenetic silencing of *miR-29* leads to differentiation arrest. The restoration of *miR-29* expression in murine models inhibits tumor growth and induces myogenic differentiation [266].

*miR-203* is also frequently silenced in RMS by promoter hypermethylation. The reactivation of *miR-203* reduces proliferation and migration while promoting terminal myogenic differentiation. Mechanistically, it exerts its tumor-suppressive effects by targeting p63 and the leukemia inhibitory factor receptor, thereby inhibiting the Notch and JAK1/STAT1/STAT3 pathways [267].

Among SNAIL-regulated miRNAs, *miR-28-3p* and *miR-193a-5p* have recently emerged as important regulators of RMS pathobiology. Both miRNAs significantly reduce proliferation and induce G0/G1 cell-cycle arrest in RMS cells. They also promote myogenic differentiation in RMS and human myoblasts by upregulating myogenic markers. In addition to their antiproliferative effects, these miRNAs impair cell migration, endothelial adhesion, chemotaxis, and invasion toward SDF-1 and HGF. They also modulate the angiogenic properties of RMS cells. In vivo, the overexpression of *miR-28-3p* and *miR-193a-5p* in xenograft models led to reduced tumor growth, impaired bone marrow colonization, and induced fibrotic tissue remodeling with aberrant vasculature. These findings identify *miR-28-3p* and *miR-193a-5p* as potential therapeutic targets in RMS [268].

A recent study highlights *miR-335-5p* as a promising biomarker in ARMS. Its expression is significantly elevated in both tumor tissues and plasma-derived extracellular vesicles (EVs) from ARMS patients, compared to ERMS and healthy controls. Its levels correlate with the Intergroup Rhabdomyosarcoma Study (IRS) stage and patient survival. In situ hybridization revealed cytoplasmic localization in malignant cells, supporting its tumor-specific expression profile [269].

Liquid biopsy studies further support the potential diagnostic and prognostic utility of circulating miRNAs. In a pilot analysis, Tombolan et al. [270] identified a specific miRNA signature in RMS patient plasma that could distinguish these patients from healthy individuals. Muscle-specific miR-206 was elevated in plasma, while potential tumor suppressors miR-26a and miR-30b/30c were significantly downregulated. Notably, low miR-26a expression was associated with *PAX3-FOXO1* fusion and adverse clinical features.

Additionally, studies have shown that *PAX3-FOXO1* alters the miRNA content of exosomes, contributing to the regulation of key oncogenic pathways. Among the affected miRNAs, *miR-486-5p* is a downstream effector of *PAX3-FOXO1*. It promotes RMS cell proliferation, invasion, and clonogenic growth and is overexpressed in both tumor cells and circulating exosomes of FP-RMS patients. The therapeutic inhibition of *miR-486-5p* in xenograft models led to decreased tumor growth, suggesting a role in ARMS progression and its potential as a therapeutic target [271].

Conversely, two miRNAs significantly downregulated in FP-RMS are *miR-221* and *miR-222*, which are co-expressed from a cluster on the X chromosome and which share several targets [272]. These miRNAs act as tumor suppressors in FP-RMS, partly by targeting *CCND2*, *CDK6*, and *ERBB3*. The overexpression of *miR-221* in FP-RMS cells induces apoptosis and reduces cell viability, motility, and invasiveness. Thus, the modulation of *miR-221/222* expression may represent an additional therapeutic avenue. Due to their ability to simultaneously regulate multiple oncogenic pathways, miRNAs like *miR-221/222* and *miR-486-5p* are emerging as attractive candidates for miRNA-based therapeutic strategies [271].

Together, these findings underscore the central role of miRNAs in ARMS biology and their promise as diagnostic, prognostic, and therapeutic tools. Ongoing and future studies are essential to validate these miRNAs as biomarkers and explore their clinical utility in RMS management.

These examples represent only a selection of the current research on miRNAs in alveolar rhabdomyosarcoma. For a broader perspective on non-coding RNAs involved in RMS pathogenesis—including microRNAs (miRNAs), long non-coding RNAs (lncRNAs), circular RNAs (circRNAs), and ribosomal RNAs (rRNAs)—readers are referred to recent comprehensive reviews [273,274,275].

This section highlights the central role of epigenetic mechanisms in ARMS pathogenesis and their emerging therapeutic potential. In FP-RMS, CHD4 and *PAX3-FOXO1* cooperatively remodel chromatin to drive oncogenic transcription, identifying BRD4 inhibition as a promising treatment strategy [39,194,202,203,204,205,206]. DNA methylation regulates gene expression and subtype identity, with patterns linked to metastatic potential. Targeting reversible methylation changes, such as *PCDHA4* silencing, offers therapeutic opportunities [195,196,197,198,199,200].

Histone modifiers—including EZH2, BMI1, KMT1A, and demethylases like KDM3A/B—maintain a proliferative state in ARMS. Their inhibition, alone or in combination with chemotherapy, shows preclinical efficacy [112,116,192,193]. Acetylation regulators, particularly KAT2B and HDACs, also support tumor growth and represent viable drug targets.

Finally, miRNAs contribute to ARMS biology and hold diagnostic and therapeutic promise, warranting further clinical validation.

## 10. Molecular Targets in ARMS—High Expectations, Limited Translation

Over the past decades, major strides have been made in decoding the molecular complexity of ARMS. This progress has unveiled a spectrum of receptor tyrosine kinases (RTKs), intracellular signaling proteins, and transcription factors that are frequently deregulated in ARMS and implicated in its pathogenesis. Among these are IGF-1R, ALK, FGFRs, EphB4, EGFR, SFKs, AKT, mTOR, and key transcriptional regulators like NF-Y. Preclinical studies have generated high expectations for targeting these molecules, often demonstrating compelling anti-tumor effects in vitro and in animal models.

However, the translation of these promising targets into successful clinical therapies has proved more difficult than anticipated. Many agents, despite early promise, have failed to show significant efficacy in clinical trials, or have faced obstacles related to compensatory signaling, limited selectivity, or adverse effects. This section critically evaluates these “high-potential” targets whose therapeutic performance has yet to meet expectations, offering a nuanced overview of their limitations, contexts of failure, and remaining opportunities. Readers interested in other RTK pathways—including FGFRs, ALK, IGF-1R, and EphB4—can also refer to the detailed discussions in Section 6.

### 10.1. IGF-1R Signaling in ARMS: A Historically Promising but Clinically Elusive Therapeutic Target

The insulin-like growth factor receptor type 1 (IGF-1R) and its ligands have been studied since the 1980s [276]. IGF-1R is a membrane-bound heterotetramer constitutively expressed by most tissues [277]. Its activation by IGF1 or IGF2 induces mitosis, protects cells against apoptosis, and helps maintain a transformed phenotype [278]. The receptor activates intracellular signaling cascades, including the PI3K/AKT and RAS/MAPK pathways, promoting cell proliferation and survival [277,279,280,281,282].

The IGF system is essential for muscle development. IGF2 stimulates skeletal myoblast proliferation and differentiation, with its expression increasing during the early stages of muscle development and decreasing upon myotube formation [283]. ARMS cells exhibit elevated levels of *IGF2*, which acts as an autocrine growth and motility factor [284]. Furthermore, IGF-1R is overexpressed in ARMS and is a transcriptional target of the *PAX3-FOXO1* oncoprotein [140,281,285]. IGF-1R is also frequently overexpressed in various human cancers, which has made it an appealing molecular target in solid tumors [276].

Preclinical models evaluating IGF-1R-targeted therapies have shown promising results. IGF-1R neutralizing antibodies [286,287,288] and small-molecule inhibitors [289,290,291] induced cell death in vitro [288,290] and tumor regression in vivo [288,290,291]. Despite these encouraging outcomes, monotherapies targeting IGF-1R have failed to show clinical efficacy. In the Phase II clinical trials (NCT00642941 [292] and NCT00831844 [293]), the monoclonal antibody cixutumumab (IMC-A12) triggered a partial response in only one of 20 ARMS patients [294]. In another study, 12 patients with relapsed or refractory ARMS were treated with the R1507 antibody. Although three of them experienced an initial tumor reduction of ≥50% at week six, they all relapsed by the next evaluation [295]. These findings suggest that while IGF-1R antibodies are generally well-tolerated, they demonstrate limited activity as monotherapies.

Given the disappointing results of single-agent therapies, research has shifted toward combination strategies. Resistance to IGF-1R inhibitors in fusion-positive (FP) and FN-RMS has been linked to increased PDGFR-β activity. The combined targeting of IGF-1R and PDGFR-β resulted in reduced cell growth in vitro and enhanced therapeutic effects in vivo in murine FN-RMS xenograft models, suggesting this as a promising strategy [296]. However, no follow-up clinical studies on this combination have been reported.

Likewise, mTOR inhibition by rapamycin paradoxically increases AKT phosphorylation. Pretreatment with the h7C10 IGF-1R antibody blocks this effect, and co-inhibition of IGF-1R and mTOR leads to the additive suppression of ARMS cell growth and survival [297]. While preclinical tests supported the efficacy of this combination [298], clinical trials (NCT01614795) [299] showed no objective responses when combining cixutumumab with temsirolimus in pediatric and young-adult patients with recurrent or refractory sarcomas [300]. Moreover, adding cixutumumab to intensive chemotherapy regimens did not improve outcomes in metastatic RMS patients [301,302].

Recent studies identified YES kinase activation as a bypass mechanism following IGF-1R inhibition. Wan et al. demonstrated that YES phosphorylation increases in response to IGF-1R blockade, suggesting that YES/SFK activation may serve as a biomarker of resistance to IGF-1R inhibitors. The combined inhibition of IGF-1R and SFKs (via dasatinib) produced enhanced anti-tumor effects in vitro and in vivo, indicating that dual targeting may yield improved therapeutic outcomes [279].

This hypothesis was clinically tested in a Phase I/II trial (NCT03041701) [303] evaluating ganitumab (AMG479), an IGF-1R antibody, in combination with dasatinib in patients with relapsed or refractory ERMS and ARMS. The Phase I portion of the study determined the maximum tolerated dose as ganitumab 18 mg/kg every two weeks combined with dasatinib 60 mg/m^2^ daily. Among nine evaluable patients, one achieved a partial response lasting four cycles, and another maintained stable disease for six cycles—reflecting a disease control rate of approximately 22% at five months. However, due to the unavailability of ganitumab, the trial was prematurely terminated, and the Phase II component was not initiated [304].

In conclusion, although IGF-1R has been recognized as a biologically significant player in ARMS and its inhibition consistently yields anti-proliferative effects in vitro and in preclinical models, clinical translation has proved challenging. Monotherapies have shown limited efficacy, and even combination strategies have failed to deliver durable responses in clinical settings. Thus, IGF-1R remains an important biological marker but is currently not a viable stand-alone therapeutic target for ARMS. Its clinical utility may be restricted to multi-targeted combination therapies that address compensatory signaling pathways.

### 10.2. ALK Signaling in ARMS: A Promising Target Undermined by Therapeutic Inconsistency

Anaplastic lymphoma kinase (ALK) is a receptor tyrosine kinase belonging to the insulin receptor superfamily. Like other classic RTKs, ALK is activated through ligand-induced homodimerization and inactivated via dephosphorylation in the absence of ligand. Upon activation, ALK triggers a range of downstream signaling cascades, including signal transducer and activator of transcription (STAT), phosphoinositide 3-kinase (PI3K)-AKT, mammalian target of rapamycin (mTOR), and mitogen-activated protein kinase (MAPK) pathways. These cascades regulate fundamental cellular processes such as growth, transformation, and survival [305]. Numerous studies have indicated that ALK expression is associated with unfavorable outcomes in rhabdomyosarcoma (RMS) [306,307,308,309]. Notably, ALK point mutations are rare in RMS [286,307], and gene amplification is absent, suggesting that ALK dysregulation may occur at the promoter level rather than through genomic alterations [307,310,311].

In an effort to improve risk stratification, Bonvini et al. proposed the quantification of *ALK* mRNA expression in RMS patients. Elevated *ALK* transcript levels were correlated with several adverse clinical features, including a PAX3/7–FOXO1-positive histology, an advanced tumor stage, and a larger tumor size at diagnosis. Furthermore, high ALK expression was associated with lower survival and increased risk of disease recurrence [308]. These findings suggest that ALK may serve as a prognostic biomarker, distinguishing both fusion-positive ARMS (FP-RMS) and aggressive fusion-negative tumors [306,308].

Based on this rationale, Bonvini et al. tested the dual ALK-MET inhibitor crizotinib—a small-molecule tyrosine kinase inhibitor originally developed for cMET and approved for *EML4-ALK*-positive non-small-cell lung cancer (NSCLC)—as a potential therapeutic agent for RMS [308]. In vitro, crizotinib completely inhibited ALK phosphorylation and activation [312]. Megiorni et al. supported these findings, reporting that crizotinib treatment led to ALK and MET inhibition, as well as the suppression of insulin-like growth factor receptor 1 (IGF-1R), the dephosphorylation of AKT and ERK, and the disruption of RMS cell growth. Treatment induced apoptosis and autophagy in a dose-dependent manner and reduced migration, invasiveness, and clonogenic potential in RH4 and RH30 cell lines [313].

Despite promising in vitro data, in vivo outcomes were less favorable. Wierdl et al. assessed RMS sensitivity to ALK inhibitors (crizotinib, NVP-TAE684, and LDK-378) and found that while RMS lines were sensitive in vitro and siRNA-mediated ALK knockdown reduced proliferation, ALK inhibition in vivo did not confer a therapeutic benefit. Alarmingly, crizotinib treatment accelerated tumor growth in patient-derived xenograft (PDX) models [309]. These preclinical findings cast doubt on ALK as a viable monotherapy target for RMS. Clinical trial results corroborated these concerns: Schöffski et al. evaluated crizotinib in patients with advanced or metastatic ARMS (NCT01524926) [314] and concluded that, although well-tolerated, the drug lacked meaningful clinical activity as a monotherapy [17].

Seeking alternative therapeutic strategies, Van Erp et al. explored the efficacy of the second-generation ALK inhibitor ceritinib in ARMS and ERMS models. Ceritinib induced cell-cycle arrest, apoptosis, and inhibited proliferation in vitro. In Rh41 xenograft models, ceritinib modestly reduced tumor growth and elevated caspase-3 levels. However, tumor regression was incomplete, and ALK signaling remained largely unaffected. The anti-proliferative effect was likely due to the suppression of IGF-1R rather than direct ALK inhibition [315]. Unfortunately, clinical trials targeting IGF-1R in RMS have yielded limited efficacy [294,295], reinforcing the notion that ALK is an unreliable standalone target for therapy.

Interestingly, Van Gaal et al. [316] identified the co-expression of IGF-1R and ALK in 68% of tested ARMS primary tumors. Motivated by this observation, they evaluated the combined effect of crizotinib and R1507 (an IGF-1R-targeting antibody) on Rh41 and Rh30 cells. Crizotinib alone inhibited growth in both cell lines, while R1507 was effective only in Rh41. The combination treatment, however, demonstrated synergistic activity against both models [316]. Despite the therapeutic rationale, no follow-up studies have yet been reported on this dual-targeting approach.

In summary, while ALK plays a role in the pathogenesis and prognosis of ARMS, preclinical and clinical data consistently suggest that it is not a reliable therapeutic target. The limited in vivo efficacy of ALK inhibitors, along with off-target effects and resistance mechanisms, underscore the challenges of targeting ALK in RMS. Combinatorial strategies, particularly those involving IGF-1R, may hold promise, but further investigation is required to validate their clinical utility.

### 10.3. EphB4 Signaling in ARMS: A Dual-Edged Target with Therapeutic Ambiguity

Ephrin receptors (Ephs) are named for their expression in erythropoietin-producing human hepatocellular carcinoma cells. Interactions between ephrins and Eph require cell-to-cell contact. They initiate bidirectional signaling downstream of the ligand-receptor complex in both the receptor-bearing cell (forward signaling) and the ligand-bearing cell (reverse signaling). Ephrins and Eph are critical in regulating cell shape, adhesion/repulsion, migration, and positioning during developmental processes that require spatially organized cell movement. The expression of ephrins and the receptors occurs during striated muscle precursor cell development, and it is still detectable in postnatal life. Alterations of ephrin-dependent mechanisms have a central role in promoting tumor progression, including ARMS [317]. ARMS tumors widely express the upregulation of ephrin-B and EphB receptors [317,318]. The indirect blocking of EphB4 forward-signaling with soluble EphB4 protein fused with murine serum albumin moderately slowed progression in ARMS orthotopic xenograft and allograft models. On the other hand, directly targeting the EphB4 through the inhibitory antibody, VasG3, failed to affect tumor progression. It seems that the inhibition of EphB4 signaling is not a viable monotherapy for ARMS [119]. Aslam et al. [318] identified EphB4 as a potential two-way switch for ARMS. They indicated that the cognitive EphB4 ligand, EphrinB2, drives tumor cells toward apoptosis. Yet, the interaction between EphB4 and another RTK, namely platelet-derived growth factor receptor beta (PDGFRβ), in the presence of the PDGFRβ ligand, PDGF-BB, leads to tumor proliferation. The simultaneous inhibition of EphB4 and PDGFRβ by dasatinib resulted in a significant decrease in cell viability and tumor growth rate and significantly prolonged survival in vivo. However, the authors admitted that the obtained results could be an effect of inhibition, not only of PDGFRβ and EphB4, because dasatinib is not selective and inhibits many tyrosine and serine-threonine kinases [318].

Ephrin-Eph signaling plays a multifaceted role in ARMS, influencing both tumor suppression and progression depending on the context of receptor-ligand interactions. While EphB4 is upregulated in ARMS and represents a potential therapeutic target, current approaches to its inhibition—either directly or indirectly—have shown limited efficacy. Notably, the dualistic function of EphB4, acting as both a promoter of apoptosis and a mediator of proliferation via interaction with PDGFRβ, underscores the complexity of targeting this pathway. Combined inhibition strategies, such as with dasatinib, show promise but lack specificity due to their broad kinase inhibition profiles. These findings highlight the need for more selective therapeutic agents and a deeper understanding of EphB4’s role within the tumor microenvironment to exploit its potential as a targeted therapy in ARMS.

### 10.4. Futibatinib: A Promising FGFR Inhibitor That Falls Short in ARMS

Although FGFR4 has emerged as a compelling therapeutic target in ARMS due to its overexpression and functional role downstream of *PAX3-FOXO1* (see Section 7.2), not all FGFR inhibitors have demonstrated clinical utility in this context.

Despite FGFR4’s therapeutic potential, not all FGFR inhibitors have proven effective in treating ARMS. One such example is futibatinib, an irreversible pan-FGFR inhibitor that targets FGFR1–4. It is FDA-approved for treating unresectable or metastatic intrahepatic cholangiocarcinoma (ICC) [319]. Preclinical research by Wu et al. [320] demonstrated that futibatinib can inhibit FGFR4 phosphorylation and reduce the proliferation of rhabdomyosarcoma (RMS) cell lines. Furthermore, combining it with chemotherapeutic agents such as irinotecan and vincristine yielded synergistic effects in vitro.

However, despite these promising in vitro results, the efficacy of futibatinib in in vivo RMS xenograft models—particularly fusion-positive (FP) ARMS—was limited. Tumors in these models showed minimal response to the drug, suggesting that futibatinib, although mechanistically rational, may not be a suitable therapeutic agent for ARMS [320]. These findings highlight the complexity of translating FGFR4-targeted therapies into clinical benefit and underscore the need for more selective and effective inhibitors tailored to the molecular landscape of ARMS.

### 10.5. EGFR Signaling in RMS: High Hopes, Low Yields

The epidermal growth factor receptor (EGFR) is involved in the development and growth of various types of malignant carcinomas [157]. Although it is overexpressed in up to 50% of ARMS tumors, it seems not to be a good target for the treatment of this particular cancer [321,322,323]. Abraham et al. [322] tested the EGFR inhibitor, Erlotinib, for its efficacy in murine model of ARMS. Erlotinib not only had no effect on tumor progression but also reduced the body weight of treated animals by 10–20%. The expression of EGFR correlates with the embryonal subtype [324], and, together with fibrillin-2, it is indicated as potential biomarkers of ERMS [57].

### 10.6. SFK–CRKL–YES Axis in RMS: A Therapeutic Prospect with Clinical Complexities

Src family kinases (SFKs) are non-receptor tyrosine kinases that regulate fundamental cellular functions such as proliferation, differentiation, apoptosis, migration, and metabolism. The vertebrate SFK family includes nine members: SRC, LCK, LYN, BLK, HCK, FYN, FGR, YES, and YRK (the latter present only in chickens) [325].

CRKs (CRK and CRKL) are adaptor proteins closely linked to SFK signaling and form part of a key signal transduction network. Acting as convergence points in tyrosine kinase pathways, they integrate signals from various sources, including growth factors, the extracellular matrix, and apoptotic cells [326].

Yeung et al. highlighted the critical role of CRKL/YES interrelated pathways in sustaining RMS cell growth and survival, suggesting the therapeutic potential of SFK inhibition in RMS [327]. Indeed, the inhibition of SRC impairs cell invasion and migration across multiple cancer types, including sarcomas [328]. SFK interference via inhibitors suppresses ARMS and ERMS cell growth in vitro and in vivo, while also identifying CRKL as a key driver of RMS progression [327].

CRKs contain Src Homology 2 (SH2) and Src Homology 3 (SH3) domains, which are separated by flexible linkers. CRKL, a 39-kDa adaptor protein with one SH2 and two SH3 domains, is significantly upregulated in RMS cell lines, xenografts, and tumor samples from both ARMS and ERMS, whereas its expression remains low in most normal tissues and other soft-tissue sarcomas [326,327].

Silencing CRKL in RMS cells induces G1-phase arrest without triggering apoptosis. This knockdown also leads to decreased phosphorylation and the activation of the YES kinase, underscoring the functional interdependence between CRKL and YES in RMS [327]. YES, a non-receptor tyrosine kinase, governs multiple cellular processes including cell growth, apoptosis, and differentiation. It contributes to cell-cycle progression by phosphorylating cyclin-dependent kinase 4 (CDK4), thus regulating the G1 phase [329,330]. YES is overexpressed at both the protein and mRNA levels in RMS tumors compared to non-RMS or normal tissues. Its knockdown impairs cell proliferation, affirming its essential role in RMS cell survival.

Yeung et al. proposed dasatinib, a known SFK inhibitor, for further exploration as a potential RMS therapy [327]. While numerous SFK-targeting drugs have reached clinical application in hematologic malignancies [331], their success in solid tumors remains limited. Experimental models have shown efficacy, yet clinical trials have revealed complications that hinder their effective use [331,332]. These challenges must be addressed to unlock the potential of SFK inhibition in treating solid tumors, including ARMS.

The SFK–CRKL–YES signaling axis plays a pivotal role in the growth and survival of RMS. Although preclinical data support the potential of SFK inhibition as a therapeutic strategy, clinical translation has been hampered by unexpected hurdles. Further research is essential to resolve these limitations and assess the viability of targeting this pathway in ARMS.

### 10.7. FAK–Src Signaling in ARMS: Synergy with Limits

Focal adhesion kinase (FAK), a nonreceptor tyrosine kinase involved in cell survival, migration, and invasion, has been implicated in RMS progression. Waters et al. demonstrated that phosphorylated FAK is expressed in ARMS tumors and cell lines. FAK inhibition, using RNAi or small-molecule inhibitors, reduced cell viability and invasion while promoting apoptosis. In vivo, FAK blockade slowed tumor growth, supporting its potential as a therapeutic target [333].

Building on this, van Erp et al. investigated co-targeting FAK and Src, another key kinase in tumor signaling. They found concurrent pFAK and pSrc expression in 35% of ARMS samples. In ARMS cell lines, FAK inhibition (defactinib) and Src inhibition (dasatinib) each reduced cell viability, while combination treatment fully suppressed both pathways, enhanced DNA damage, and showed synergistic cytotoxicity.

However, this synergy did not translate in vivo, as combined treatment failed to significantly reduce tumor growth in preclinical models compared to dasatinib alone [315]. These findings underscore the therapeutic potential of FAK-Src targeting in ARMS, while highlighting the challenge of translating in vitro success into in vivo efficacy.

### 10.8. AKT Signaling in ARMS: A Double-Edged Sword

Protein kinase B (PKB/AKT) has emerged as a central regulator of the PI3K–AKT–mTOR signaling axis, a pathway frequently implicated in tumorigenesis due to its roles in promoting cell growth, survival, and proliferation. In many cancers, constitutive activation of this pathway—often resulting from upstream mutations—supports uncontrolled cell division and resistance to apoptosis, positioning AKT as an attractive therapeutic target.

In the context of ARMS, AKT has generated considerable interest due to its complex and somewhat paradoxical function. On the one hand, the activation of AKT has been shown to antagonize the oncogenic transcriptional activity of the *PAX3-FOXO1* fusion protein. Jothi et al. demonstrated that pharmacologically induced AKT activation leads to the phosphorylation-dependent inhibition of *PAX3-FOXO1*, the suppression of tumor-cell growth and invasiveness, and the induction of apoptosis in vitro, along with reduced tumor burden in xenograft models [334,335]. These findings gave rise to the hypothesis that transient AKT activation could serve as a form of differentiation therapy, especially since *PAX3-FOXO1* inhibition is known to restore terminal myogenic differentiation [49].

Further mechanistic insights suggest that AKT negatively regulates myogenic gene expression through its interaction with *PAX3-FOXO1*, thereby contributing to the differentiation blockade characteristic of ARMS [334]. This raised high expectations that modulating AKT signaling—either through activation or inhibition—could serve as a viable therapeutic strategy.

However, the clinical translational potential remains uncertain. Shen et al. [336] reported that AKT inhibition using perifosine significantly impaired ARMS cell viability and tumor growth in vivo [336]. Yet, their findings also indicated that the antitumor effects of perifosine are likely mediated by additional mechanisms, suggesting that *AKT1* may not be the sole or primary target.

This duality—where both the activation and inhibition of AKT appear to yield antitumor effects depending on cellular context—highlights the inherent complexity of targeting this pathway in ARMS. Despite encouraging preclinical results, no AKT-targeted therapies have yet advanced to clinical application in rhabdomyosarcoma. Moreover, the risk of unintended effects on normal tissues and the broader role of *AKT1* in cellular homeostasis further complicate its therapeutic exploitation.

In conclusion, while AKT remains a biologically significant molecule with clear mechanistic links to *PAX3-FOXO1*-driven oncogenesis, the path toward its clinical targeting in ARMS is fraught with uncertainty. The high expectations generated by early studies have thus far translated into limited therapeutic perspectives, underscoring the need for more nuanced, context-specific strategies in future research.

### 10.9. mTOR Signaling in ARMS: Therapeutic Promises and Limitations

mTOR (mammalian target of rapamycin) is involved in many signaling pathways, regulating cell proliferation, autophagy, and apoptosis, and it plays a critical role in tumor metabolism and invasion downstream of AKT [337]. In preclinical RMS studies, mTOR inhibition has reduced cellular migration, invasion, tumor growth, and angiogenesis [133,338,339], suggesting that targeting mTOR could be therapeutically useful. In ARMS, the activation of the PI3K/AKT/mTOR pathway correlates with poor survival, with increased phosphorylation of its components observed in tumors from patients with reduced disease-free or overall survival. For example, although the ATP-competitive mTOR inhibitor AZD8055 showed broad in vitro activity, its in vivo performance in ARMS was limited to disease progression without objective tumor regression [340]. Conversely, mTOR inhibition can sensitize ARMS cells to apoptosis induced by other agents, as demonstrated by the significant enhancement of ABT-737-induced apoptosis in ARMS cells when co-treated with AZD8055, despite the minimal cytotoxicity of AZD8055 alone [341]. Similarly, temsirolimus (CCI-779) suppressed downstream protein phosphorylation and reduced tumor growth in ARMS xenografts via an antiangiogenic mechanism linked to mTOR/HIF-1α/VEGF signaling [338]; however, clinical trials, such as the Phase III Children’s Oncology Group ARST1431 trial, showed that adding temsirolimus to chemotherapy did not improve event-free survival in intermediate-risk RMS patients [342], highlighting the need for novel, biology-based combination strategies.

RMS frequently exhibits co-activation of the PI3K/AKT/mTOR and MAPK pathways, with PI3K active in over 80% of cases and frequent MAPK co-activation. The inhibition of one pathway often leads to the compensatory activation of the other, contributing to resistance against monotherapies. This is evidenced by the synergistic suppression of tumor growth and the effective inhibition of phosphorylated AKT, ERK, and S6 achieved with combined treatment of the TORC1/2 inhibitor AZD8055 and the MEK inhibitor AZD6244, whereas the combination of the PI3K/mTOR inhibitor NVP-BEZ235 with AZD6244 showed limited efficacy [343]. In ARMS, the dual inhibition of PI3K/mTOR and MEK pathways with PI103 and UO126 induces synthetic lethality and robust apoptosis (CI < 0.1). Co-silencing of *PIK3CA* (p110α) and *MAP2K1/MAP2K2* (MEK1*/2*) further confirms the cooperative effect. Mechanistically, this combination overcomes compensatory pathway activation—PI103 alone increases ERK phosphorylation, and UO126 alone enhances Akt activation—while downregulating anti-apoptotic proteins such as XIAP, Bcl-xL, and Mcl-1 [344].

A further strategy involves dual inhibition of the Hedgehog (HH) and PI3K/AKT/mTOR pathways. The combination of the GLI1/2 inhibitor GANT61 and the PI3K/mTOR inhibitor PI103 synergistically induces apoptosis in RMS cell lines and primary cells (CI < 0.2) and suppresses clonogenicity, three-dimensional growth, and in vivo tumor progression. Mechanistically, this co-treatment triggers mitochondrial, caspase-dependent apoptosis via BAK/BAX activation and the upregulation of pro-apoptotic proteins NOXA and BMF, with resistance conferred by the knockdown of these proteins or the overexpression of *BCL-2/MCL-1* [345].

Collectively, these data indicate that, while mTOR is a pivotal regulator of tumor metabolism, invasion, and angiogenesis in ARMS, inhibition of mTOR alone is insufficient due to compensatory activation of parallel signaling pathways. Combination strategies targeting both mTOR and its compensatory mechanisms—such as the co-inhibition of PI3K/mTOR and MEK or Hedgehog pathways—offer greater therapeutic potential. However, the complexity of these signaling networks and the challenges observed in clinical translation underscore the need for further innovative, biology-based approaches to improve outcomes in ARMS patients.

### 10.10. NF-Y in ARMS: An Indispensable Oncogenic Driver with Unavoidable Pitfalls

NF-Y is a conserved, sequence-specific trimeric transcription factor that binds the CCAAT element in gene promoters, consisting of the subunits NF-YA, NF-YB, and NF-YC, each essential for its role in DNA binding and function. It regulates key biological processes such as cellular proliferation, apoptosis, oncogenesis, and stress responses [346]. In ARMS, phenotypic screening has revealed that NF-Y inhibits myogenic differentiation in concert with the *PAX3-FOXO1* fusion protein. Loss of any NF-Y subunit produces a phenotype similar to *PAX3-FOXO1* inactivation. While NF-Y and *PAX3-FOXO1* display overlapping transcriptomic profiles in RMS cells, NF-Y primarily occupies promoter regions, in contrast to the distal enhancer binding of *PAX3-FOXO1*. Notably, NF-Y directly activates *PAX3-FOXO1* expression by binding to CCAAT motifs upstream of the *PAX3* gene, thereby enhancing oncogenic signaling through the activation of cell-cycle and metabolism-related genes. Given its critical role in sustaining the malignant phenotype in ARMS, NF-Y emerges as a potential target for differentiation therapies; however, its essential functions in normal tissues present significant challenges and raise concerns over adverse effects [347].

### 10.11. PODXL in FP-RMS: Promising Yet Risk-Prone

PODXL is a transmembrane glycoprotein that plays a crucial role in mediating cell adhesion and it is actively involved in the metastatic dissemination of tumor cells. It facilitates tumor growth, invasion, and metastasis, with its overexpression reported in various cancer types [111,348]. In fusion-positive rhabdomyosarcoma (FP-RMS) tumors, PODXL is notably upregulated, implicating it as a key driver of cancer progression. Functional studies have demonstrated that reducing *PODXL* expression not only diminishes colony formation but also significantly impairs the metastatic potential of tumor cells [111]. These findings suggest that PODXL contributes substantially to the aggressive phenotype observed in FP-RMS, thereby positioning it as a potential target for therapeutic intervention.

However, the expression of PODXL in certain normal tissues poses a significant challenge; targeting this glycoprotein could lead to on-target, off-tumor effects, raising concerns about potential adverse side effects in non-cancerous cells [348]. Consequently, while PODXL represents a promising target for disrupting tumor progression, careful consideration must be given to strategies that can selectively inhibit its function in tumor cells without affecting its normal physiological roles.

While the molecular characterization of ARMS has identified a variety of potentially druggable targets—including IGF-1R, ALK, EphB4, EGFR, and several intracellular kinases—their therapeutic efficacy has often fallen short of expectations. Failures in clinical translation, whether due to tumor heterogeneity, pathway redundancy, or compensatory mechanisms, have underscored the complexity of ARMS biology and the limitations of monotherapies. A summary of the molecular targets, inhibitors, and their therapeutic outcomes discussed in this section is presented in Table 3.

Despite these challenges, the exploration of combinatorial strategies, multi-target inhibitors, and context-specific interventions offers renewed hope. Importantly, understanding why certain therapies fail can be just as instructive as discovering new targets. Future therapeutic advances in ARMS will depend not only on innovative drug development but also on the integration of predictive biomarkers, improved preclinical models, and systems biology approaches that reflect the dynamic nature of tumor-signaling networks. Until then, many promising targets remain therapeutic enigmas—worthy of further study, yet still awaiting effective clinical realization.

### 10.12. Lessons from Failed Monotherapies: Implications for Rational Combination Strategies

In fusion-positive rhabdomyosarcoma (FP-RMS), various targeted therapies have yielded only brief or modest benefits in trials. IGF-1R inhibitors, such as monoclonal antibodies, showed minimal activity—a Phase 2 study in relapsed RMS reported only one confirmed partial response among 36 patients [295] and adding an anti-IGF-1R antibody to upfront chemotherapy failed to improve outcomes [349,350]. The limited efficacy is attributed to the lack of biomarker-guided selection and adaptive resistance via bypass signaling (e.g., through YES/SRC or ALK kinases) [288,351].

CDK4/6 inhibitors have also underperformed: in a recent pediatric basket trial of *CDK4/6*-altered tumors (including FP-RMS), palbociclib monotherapy elicited no objective responses [352], even in tumors harboring pathway amplification. Preclinical data suggest that FP-RMS cells rapidly compensate via PI3K/AKT or MAPK signaling, limiting the durability of CDK4/6-targeted responses [353].

BET bromodomain inhibitors, which interfere with the BRD4-mediated regulation of *PAX3-FOXO1*, show potent preclinical efficacy—for example, JQ1 downregulates the fusion protein and its transcriptional program [39] However, clinical progress has been limited by hematologic toxicities (notably thrombocytopenia) that constrain effective dosing [354,355].

Similarly, Aurora A kinase inhibitors like alisertib have demonstrated modest clinical activity: pediatric trials reported low response rates and frequent myelosuppression [356]. While Aurora A inhibition can reduce *PAX3-FOXO1* and MYCN levels, it typically induces growth arrest rather than apoptosis. Combinatorial strategies, such as co-targeting anti-apoptotic proteins (e.g., BCL2) alongside Aurora A, have shown synergistic efficacy in FP-RMS models [72].

Together, these findings highlight that monotherapies targeting single vulnerabilities in FP-RMS are insufficient. Tumor plasticity, signaling redundancy, and the absence of biomarker-based stratification have undermined translational success. These lessons are now guiding a shift toward rational combination regimens and molecularly selected patient cohorts, which may be necessary to achieve sustained clinical responses [350].

## 11. Emerging Tools for Target Discovery and Resistance Monitoring: Liquid Biopsy and CRISPR Screens

Technologies such as liquid biopsy and CRISPR-based functional screening are gaining traction in fusion-positive rhabdomyosarcoma (FP-RMS) as tools to guide precision medicine. These approaches offer noninvasive molecular monitoring and systematic target identification that can refine treatment design and reveal resistance pathways.

Liquid biopsy, particularly using circulating tumor DNA (ctDNA), enables real-time detection of fusion transcripts and resistance-associated mutations in blood. Recent studies have successfully identified the hallmark *PAX3-FOXO1* fusion in plasma samples from FP-RMS patients using next-generation sequencing or digital PCR, with detection rates of 86–100% in relapsed or high-burden cases [357,358,359]. These assays also detect emerging alterations (e.g., *RAS*, *PIK3CA*, *CDK4* amplifications) that may inform therapeutic resistance or relapse. Although detection in localized tumors remains challenging due to low DNA shedding, liquid biopsy shows promise for longitudinal surveillance and early relapse prediction.

CRISPR-based functional genomic screens are uncovering key molecular dependencies in FP-RMS. For example, whole-genome CRISPR knockout screening identified the NF-Y transcription factor complex as essential for sustaining *PAX3-FOXO1* expression and blocking differentiation in FP-RMS models. The disruption of NF-Y triggered spontaneous myogenic reprogramming [347]. Separately, metabolic CRISPR screens revealed that *PAX3-FOXO1*-positive cells are vulnerable to dihydrofolate reductase (DHFR) inhibition via methotrexate [360]. Other screens uncovered FOS/AP-1-mediated resistance to ATR inhibitors, pointing to combination strategies that suppress DNA damage responses and escape signaling [94].

Together, these technologies offer powerful tools to refine patient stratification, monitor therapeutic responses, and uncover hidden therapeutic vulnerabilities in FP-RMS. Their integration into early-phase clinical trials may accelerate progress toward biomarker-driven treatment strategies for this aggressive pediatric cancer.

## 12. Conclusions

Fusion-positive alveolar rhabdomyosarcoma (FP-RMS) remains one of the most aggressive pediatric soft tissue sarcomas, with limited improvement in survival outcomes for patients with advanced or metastatic disease. Despite standard multimodal therapies significantly improving prognosis in localized RMS, the five-year overall survival for high-risk FP-RMS remains below 30% [1].

The increasing understanding of ARMS biology has highlighted multiple molecular vulnerabilities, particularly those driven by the *PAX3-FOXO1* fusion oncoprotein, which reprograms the transcriptome and contributes to proliferation, survival, and blocked differentiation [2,3]. Therapeutic strategies targeting *PAX3-FOXO1* directly remain elusive; however, indirect approaches—including the inhibition of downstream effectors such as *FOXF1*, IGF-1R, MET, CDK4, and ATR—are being actively explored [1,3,4,5].

Tyrosine kinase inhibitors (TKIs), such as anlotinib and FGFR/PDGFR inhibitors, have shown preclinical efficacy in ARMS models but frequently fall short in clinical trials, largely due to tumor heterogeneity, pathway redundancy, and compensatory survival mechanisms [5,6,7]. Similarly, transcription factors such as TBX2, MYOGENIN, FOXF1, ETS1, and SNAIL represent promising but challenging therapeutic targets due to their lack of enzymatic domains and nuclear localization [8,9,10,11].

Recent investigations have also brought attention to epigenetic modulators and non-canonical signaling mediators (e.g., TRIB3 pseudokinase, Staufen1, and RNA-binding proteins), which influence ARMS pathogenesis and may represent novel intervention points [4,12].

While many experimental therapies—ranging from kinase inhibition and epigenetic modulation to immune-based strategies—have demonstrated potential in vitro or in xenograft models, translation into clinically effective treatments remains limited. The lack of durable responses to monotherapies in ARMS underscores the urgent need for rationally designed combination regimens, informed by predictive biomarkers and molecular profiling [8,13,361].

In conclusion, although progress in the molecular understanding of ARMS has expanded the repertoire of potential therapeutic targets, the development of effective, selective, and clinically viable strategies is still in its infancy. Future efforts must focus on biomarker-driven trials, better patient stratification, and integrative multi-target approaches to improve outcomes for children and adolescents affected by this highly malignant disease.

## Figures and Tables

**Table 1:** Key molecular alterations and potential therapeutic targets in alveolar rhabdomyosarcoma (ARMS).

Molecular Feature	Gene/Protein	Alteration Type	Functional Impact	Therapeutic Implication	Ref.
Fusion oncogene	*PAX3-FOXO1/PAX7-FOXO1*	Gene fusion (t(2;13), t(1;13))	Potent transcriptional activator, pioneer factor, super-enhancer rewiring	Core oncogenic driver; indirect targeting via co-factors like BRD4	[12,13,19,20,21,29,38,39]
Cell cycle regulator	*CDK4*	Amplification (12q13–q14)	Promotes cell cycle progression	CDK4/6 inhibitors (e.g., palbociclib)	[22]
Tumor suppressor	*CAV1*	Downregulation/silencing	Loss of signaling modulation	Potential diagnostic marker	[23]
Oncogene cooperation	*CDKN2A*, *TP53*	Deletion, mutation, promoter methylation	Loss of tumor suppressor function (p16, p53)	Potential sensitization to DNA-damaging agents	[33,34,35,36]
RTK signaling	*FGFR4*, *IGF1R*, *ALK*, *MET*, etc.	Overexpression, mutation	Altered proliferative signaling	Targetable RTKs	[24,36]
Epigenetic regulator	BRD4	Functional dependency via SE recruitment	Maintains transcriptional program driven by *PAX3-FOXO1*	BET inhibitors (e.g., JQ1)	[39]
Transcription factors	*MYOD1*, *MYCN*, *MYOG*	Amplification, mutation	Cooperative activation of muscle-lineage genes	Targetable dependencies in SE networks	[37,38]
Signaling pathways	PI3K, RAS	Pathway activation (mutation, amplification)	Enhanced survival and proliferation	PI3K/mTOR or RAS/MAPK inhibitors	[14,24,37,41]
Tumor suppressor targets	*DAPK1*, *GREM1*, *HEY1*	Altered expression via *PAX3-FOXO1*	Suppressed apoptosis, altered differentiation	Indirect targeting possible	[30]
Core regulatory TFs (CR TFs)	*MYOD1*, *MYOG*, *MYCN*	SE-driven overexpression	Define oncogenic transcriptional circuitry	Epigenetic therapy, CR TF network disruption	[42]

**Table 2:** RTKs and their inhibitors in fusion-positive rhabdomyosarcoma.

RTK	Role in FP-RMS	Key Inhibitors	Clinical Status	Source
FGFR4	Overexpressed; transcriptional target of *PAX3-FOXO1*; promotes proliferation	FGF401, INCB062079	Preclinical and early-phase trials in FGFR4-driven tumors	[127]
IGF1R	Activates PI3K/AKT signaling; supports cell survival and proliferation	Linsitinib (OSI-906), Xentuzumab	Phase I/II trials; limited efficacy in FP-RMS	[128]
PDGFRα/β	Enhances tumor growth through autocrine signaling loops	Imatinib, Sunitinib	Preclinical and early trials in RMS; not approved	[41]
MET	Direct *PAX3-FOXO1* target; promotes cell motility and invasion	Crizotinib, Tivantinib	Phase I/II trials; limited clinical benefit in RMS	[17]
ALK	Aberrant expression; associated with resistance and poor prognosis	Ceritinib, Lorlatinib	Explored in pediatric tumors; limited RMS-specific data	[129]
EGFR	Occasionally activated; may cooperate with IGF1R signaling	Erlotinib, Gefitinib	Explored in sarcoma; limited data in RMS	[130]
VEGFR1/2	Supports angiogenesis and tumor–stroma interactions	Bevacizumab, Axitinib	Studied in soft tissue sarcomas; no RMS-specific results	[131]

**Table 3:** Summary of selected molecular targets and inhibitors evaluated in ARMS, their biological functions, therapeutic approaches, and limitations.

Target	Biological Role	Mechanism in ARMS	Therapeutics Investigated	Preclinical Results	Clinical Outcome
IGF-1R	RTK involved in growth, proliferation, survival	Overexpressed in ARMS; transcriptional target of *PAX3-FOXO1*	Cixutumumab, R1507, AMG479 (Ganitumab)	Effective in vitro and in xenografts; synergistic with mTOR/SFK inhibitors	Limited efficacy in trials (NCT00642941, NCT00831844); combination trials ongoing (NCT03041701)
ALK	RTK from insulin receptor family; regulates growth signaling	Overexpressed in high-risk RMS; no gene amplification or activating mutations	Crizotinib, Ceritinib	Crizotinib effective in vitro; Ceritinib effective in Rh41 model	Crizotinib accelerated tumor growth in PDX; limited activity in clinical trial (NCT01524926)
EphB4	RTK; regulates cell positioning and vasculature	Overexpressed in ARMS; dual role in apoptosis and proliferation via PDGFRβ	Dasatinib, VasG3 antibody, soluble EphB4	Dual inhibition of EphB4 and PDGFRβ reduced growth and improved survival	Therapeutic ambiguity; direct inhibition ineffective
Futibatinib	Pan-FGFR irreversible inhibitor	Inhibits FGFR1–4 signaling; targets FGFR4, which is upregulated in ARMS	Futibatinib	Inhibits RMS cell proliferation in vitro; synergy with chemotherapy	Poor response in xenograft models; not effective in FP-RMS
EGFR	RTK involved in cell proliferation and survival	Expressed in RMS; unclear dependency	Erlotinib, Gefitinib	Some in vitro activity; enhanced by IGF-1R inhibition	No clinical success reported
SFK–CRKL–YES	Non-receptor tyrosine kinase cascade	YES activation bypasses IGF-1R inhibition; CRKL regulates RTK signaling	Dasatinib	Dual inhibition with IGF-1R antibody enhanced response	Phase I/II trial in progress (NCT03041701)
FAK–Src	Cytoplasmic kinases involved in adhesion, migration, and survival	Hyperactivated in ARMS; promotes invasiveness	FAK inhibitors + Dasatinib	Combination showed synergy in reducing motility and invasion	Clinical data not available
AKT	Central node in PI3K signaling; regulates survival and metabolism	Hyperactivation supports cell survival; regulated by IGF-1R, TRIB3	AKT inhibitors (various)	Inhibition reduces proliferation; affected by multiple feedback loops	Not evaluated as monotherapy in ARMS clinical trials
mTOR	Downstream kinase of PI3K/AKT; regulates protein synthesis	Activated in ARMS; promotes growth and resistance	Temsirolimus + Cixutumumab	Effective in vitro and xenografts; synergistic with IGF-1R inhibitors	Failed to produce objective responses in Phase II (NCT01614795)
NF-Y	Transcription factor controlling cell cycle genes	Supports *PAX3-FOXO1* expression; controls growth and survival	siRNA, potential future inhibitors	NF-Y inhibition reduces tumor cell proliferation and *PAX3-FOXO1* expression	Not yet explored in clinical settings
PODXL	Anti-adhesive transmembrane protein	Direct target of *PAX3-FOXO1*; supports migration, invasion, drug resistance	Knockdown models	Depletion impairs ARMS cell growth and survival	No clinical inhibitors; promising target for further research

## Abbreviations

The following abbreviations are used in this manuscript:

ARMS	Alveolar Rhabdomyosarcoma
CAR T	Chimeric Antigen Receptor T-Cells
CR TFs	Core Regulatory Transcription Factors
CRISPR	Clustered Regularly Interspaced Short Palindromic Repeats
ERMS	Embryonal Rhabdomyosarcoma
FN-RMS	Fusion-Negative Rhabdomyosarcoma
FP-RMS	Fusion-Positive Rhabdomyosarcoma
HSMM	Human Skeletal Muscle Myoblasts
IAPs	Inhibitor of Apoptosis Proteins
IHC	Immunohistochemistry
IVA	Ifosfamide, Vincristine, Actinomycin D
MTD	Maximum Tolerated Dose
PK	Pharmacokinetics
PPI	Protein–Protein Interaction
PRMS	Pleomorphic Rhabdomyosarcoma
RMS	Rhabdomyosarcoma
RP2D	Recommended Phase 2 Dose
SD	Stable Disease
SE	Super-Enhancer
SRMS	Spindle Cell/Sclerosing Rhabdomyosarcoma
VAC	Vincristine, Actinomycin D, Cyclophosphamide
sdAb	Single-Domain Antibody
siRNA	Small Interfering RNA
